# Asymmetric Triphenylethylene-Based Hole Transporting Materials for Highly Efficient Perovskite Solar Cells

**DOI:** 10.1021/acsami.3c17811

**Published:** 2024-02-06

**Authors:** Julius Petrulevicius, Yi Yang, Cheng Liu, Matas Steponaitis, Egidijus Kamarauskas, Maryte Daskeviciene, Abdulaziz S. R. Bati, Tadas Malinauskas, Vygintas Jankauskas, Kasparas Rakstys, Mercouri G. Kanatzidis, Edward H. Sargent, Vytautas Getautis

**Affiliations:** † Department of Organic Chemistry, Kaunas University of Technology, Radvilenu pl. 19, Kaunas 50254, Lithuania; ‡ Department of Chemistry, Northwestern University, 2145 Sheridan Rd, Evanston, Illinois 60208, United States; § Department of Electrical and Computer Engineering, Northwestern University, 2145 Sheridan Rd, Evanston, Illinois 60208, United States; ∥ Institute of Chemical Physics Vilnius University, Sauletekio al. 3, Vilnius 10257, Lithuania

**Keywords:** hole-transporting material, triphenylethylene, high efficiency, perovskite, solar cells

## Abstract

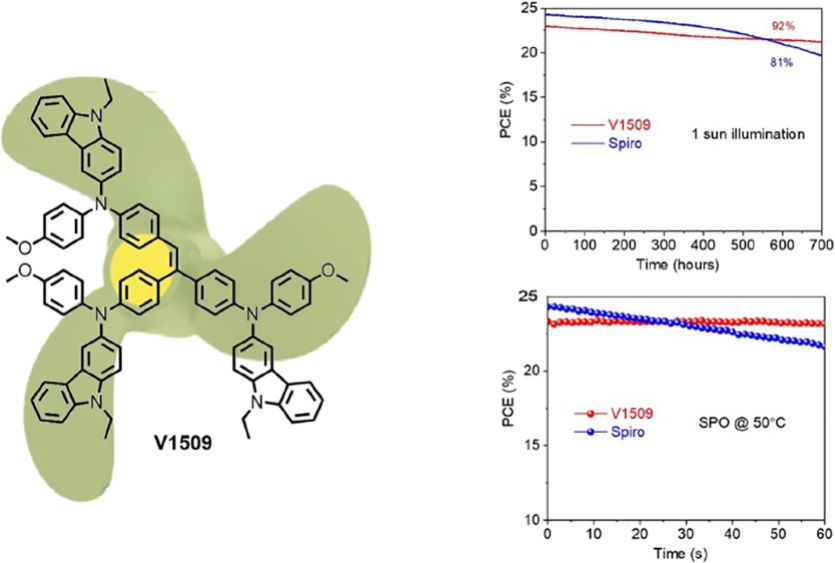

Molecular hole-transporting materials (HTMs) having triphenylethylene central core were designed, synthesized, and employed in perovskite solar cell (PSC) devices. The synthesized HTM derivatives were obtained in a two- or three-step synthetic procedure, and their characteristics were analyzed by various thermoanalytical, optical, photophysical, and photovoltaic techniques. The most efficient PSC device recorded a 23.43% power conversion efficiency. Furthermore, the longevity of the device employing V1509 HTM surpassed that of PSC with state-of-art spiro-OMeTAD as the reference HTM.

## 1 Introduction

In the past decade, perovskite solar cells (PSCs) have proved to be a promising alternative for conventional silicon photovoltaics due to their low cost, high power conversion efficiency (PCE) (nearly 26%), and ease of device fabrication.^1−6^ The rapid development of PSCs does not guarantee that the technology is ready for the market just yet; there are some issues that need to be addressed before mass production. Two of the main problems that need to be solved are the limited stability of charge-transporting materials, and the longevity of the perovskite composition and devices.^7,8^ An important component of the PSC is the hole-transporting layer (HTL) which ensures that the holes efficiently travel from the absorber toward the electrode. To ensure this, the HTL has to meet certain requirements: chemical and morphological stability, appropriate energy levels, and high carrier mobility.^9^ At the moment, the highest recorded PCE was achieved with either 2,2′,7,7′-tetrakis(*N,N*-di-*p*-methoxyphenylamine)-9,9′-spirobifluorene (spiro-MeOTAD) or poly[bis(4-phenyl)(2,4,6-trimethylphenyl)amine] (PTAA) as a HTL; however, both pose problems of their own due to relatively expensive and difficult synthesis, while spiro-MeOTAD also suffers from stability issues.^8,10−12^ As a result, several new molecular classes have been developed based on different strategies. One popular approach to create efficient hole-transporting materials (HTMs) involves modifying the substituents around the central core of spiro or a similar core. Following this approach, HTMs such as X55, SCZF-5, and SFXDAnCBZ have been synthesized, demonstrating PCE of 20.8, 20.1, and 20.87%, respectively.^13−15^ Although this strategy has shown promising results in terms of achieving a high PCE, it is important to acknowledge its limitations. One significant drawback is the cost and complexity associated with synthesizing the central core used in these materials.

An alternative and optimized approach for designing a HTM involves using a less complex central fragment, while incorporating substituents known for their strong electron-donating properties. These substituents can be selected adjusting the highest occupied molecular orbital (HOMO) level in the desired direction, ultimately leading to high PCE. For instance, carbazole derivatives have proven successful in creating various HTMs that exhibit highly efficient performance in PSCs.^16^^−20^ As for the central core, there are many viable options described in the literature, e.g., tetraphenylethylene, triphenylamine, thieno[3,4-*b*]pyrazine, triazatruxene etc. were all used to synthesize in the well-performing HTMs;^21−31^ however, triphenylethylene could be an especially interesting choice due to its asymmetric propeller shape that could theoretically form a three-dimensional charge-transporting network, which in turn should improve a good performance of hole transport as stated in the study of Chen et al.^32^

In this study, we demonstrate the application and synthesis of new HTMs based on a triphenylethylene central moiety and carbazole donors as substituents. Said HTMs are synthesized under a simple two- or three-step synthetic scheme. Next, we thoroughly documented their thermal, optical, and photoelectrical properties. The new materials were applied as organic p-type semiconductors and successfully applied in PSCs, reaching efficiencies of above 23%. Furthermore, the device based on the champion HTM V1509 compound exhibited increased stability once compared to PSCs with spiro-MeOTAD.

## 2 Results and Discussion

**Scheme 1:**
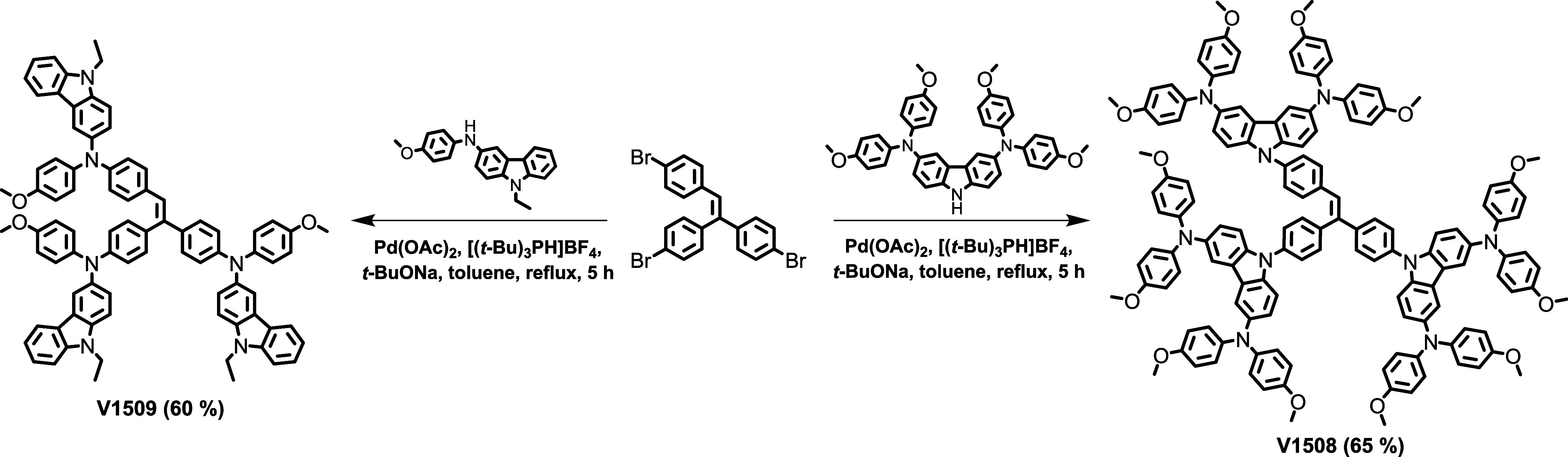
Synthesis of HTMs V1508 and V1509

Triphenylethylene-based materials V1508 and V1509 were synthesized by palladium cross-coupling reactions between 4,4′,4″-(ethene-1,1,2-triyl)tris(bromobenzene) and respective carbazole derivatives (Scheme 1). Detailed synthetic procedures of novel HTMs are described in the Supporting Information.

**Figure 1:**
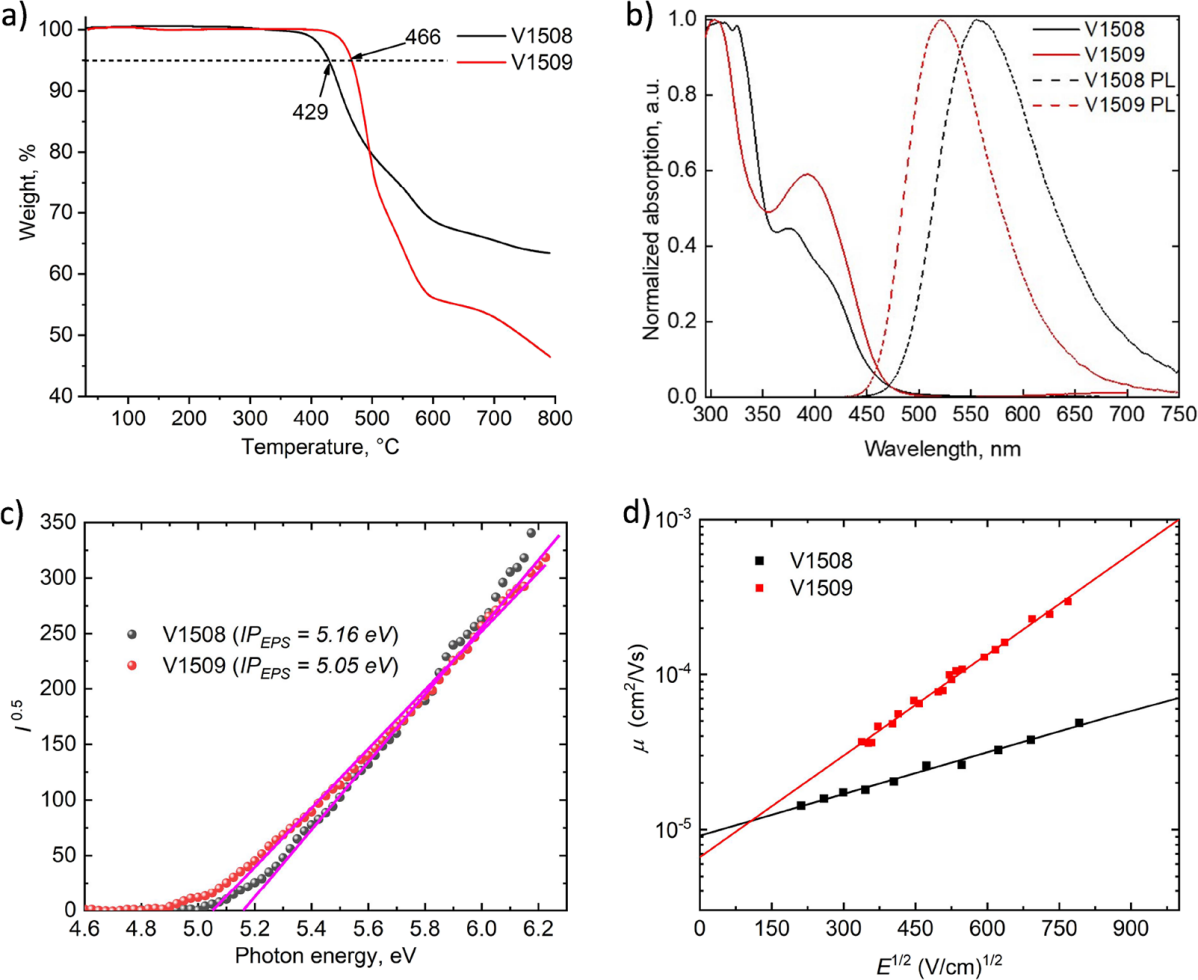
(a) TGA curves of V1508 and V1509; (b) solid-state UV–vis absorption (solid line) and photoluminescence (dashed line) spectra of V1508 and V1509 in the solid state; (c) ionization potential; (d) hole-drift mobility of V1508 and V1509.

To determine thermal and morphological stability, thermal gravimetric analysis (TGA) and differential scanning calorimetry (DSC) were used. TGA results show that both HTMs are sufficiently stable with decomposition temperatures above 400 °C (Figure 1a) which is even more than required for conventional device operation.^7^ Analysis of DSC results reveals that triphenylethenyl derivative V1508 is a molecular glass with a glass transition temperature (*T*_g_) of 233 °C, while V1509 is also amorphous with a lower *T*_g_ of 148 °C (Figure S1), the difference in *T*_g_ could be explained by the bulkier structure of V1508 compared to V1509. Nevertheless, both materials demonstrated *T*_g_ higher than that of spiro-MeOTAD (124 °C)^33^ suggesting that newly synthesized HTMs are morphologically more stable.

The UV–vis absorption and photoluminescence (PL) spectra of the new triphenylethylene derivatives V1508 and V1509 can be seen in Figure 1b. Both materials have similar absorption spectra: they absorb light most intensively at ∼300 nm, which corresponds to the π–π* electron transition, while less intense peaks that may be attributed to *n*–π* transitions are recorded at roughly 380 nm for V1508 and around 400 nm for V1509. The PL spectra of the new organic semiconductors are normalized at peak values of 556 nm for V1508 and 521 nm for V1509. Interestingly, V1508 has a higher Stokes shift of 179 nm in comparison to the 128 nm of V1509 which could be explained by the larger size of V1508 resulting in a higher energy loss by nonradiative ways. The optical gaps (*E*_g_) were calculated from the intersection of absorption and PL spectra and were found to be 2.6–2.7 eV (Table 1).

**Table 1:** Thermal, Optical, and Photophysical Properties of the Synthesized Materials

ID	*T*_g_, °C^a^	*T*_dec_, °C^a^	λ_abs_, nm^b^	λ_em_, nm^c^	*I*_p_, eV^d^	*E*_g_, eV^e^	*E*_ea_, eV^f^	μ_0_, cm^2^ V^–1^ s^–1^^g^
V1508	233	429	307, 375	556	5.16	2.62	2.54	9.1 × 10^–6^
V1509	148	466	304, 393	521	5.05	2.69	2.36	6.6 × 10^–6^

a Glass transition (*T*_g_) and decomposition (*T*_dec_) temperatures were determined by DSC and TGA, respectively (10 °C/min, N_2_ atmosphere).

b Absorption spectra measured in the solid state.

c Emission spectra measured from thin films.

d Ionization potentials of thin films measured using PESA.

e *E*_g_ estimated from the intersection of absorption and emission spectra.

f *E*_ea_ = *I*_p_ – *E*_g_.

g Mobility value of undoped materials at zero field strength.

To evaluate the compatibility of the new HTMs with the perovskite, the ionization potential (*I*_p_) was evaluated by the photoelectron spectroscopy in the air (PESA) (Figure 1c). From the results, triphenylethylene derivatives V1508 and V1509 possess the necessary energy levels needed for the transfer of holes from the perovskite toward the electrode (Table 1). It is also worth noting that although both HTMs have suitable energy levels, V1508 has a slightly higher *I*_p_ value (by 0.11 eV) which might result from more steric hindrance occurring due to the larger molecule size compared to V1509. In addition, to understand the effect of the p-dopants, ionization potentials of doped layers have been measured. Upon doping, ionization potentials were stabilized by ∼0.3 eV for V1509 and spiro-OMeTAD^34^ and 0.1 eV for V1508, respectively, to further reduce the overpotential with the valence band of the perovskite (Figure S10). Furthermore, based on *I*_p_ and *E*_g_ values, we calculated the electron affinity (*E*_ea_) in the range of 2.35–2.55 eV, these values are acceptable for efficient electron blocking in PSCs.^35^

The hole mobility of undoped materials was revealed by using the xerographic time-of-flight with the electric field dependencies of the hole-drift mobility shown in Figure 1d. Mobility at zero field strength (μ_0_) of new HTMs V1508 and V1509 was measured to be 9.1 × 10^–6^ and 6.6 × 10^–6^ cm^2^ V^–1^ s^–1^, respectively, demonstrating that in this case, the size of substituents around triphenylethylene has little effect on the speed of charge extraction.

To investigate the hole extraction ability of the newly synthesized HTMs in PSCs, we fabricated n-i-p PSCs devices with the following structure: fluorine-doped tin oxide (FTO)/SnO_2_/perovskite/phenylethylammonium iodide (PEAI)/HTM/Au. The perovskite composition was FA_0.85_MA_0.1_Cs_0.05_PbI_3_ (FA: formamidinium, MA: methylammonium) with 32 mol % methylammonium chloride as the additive. We tested different concentrations of V1508 and V1509 HTMs and compared them to spiro-MeOTAD.

The PCE statistics are presented in Figure S11. We found that the optimal concentration of V1508 was 80 mg/mL, which resulted in an average PCE of 21%. Since this concentration was the saturated solution, we could not determine the precise concentration, and the filtered solution was used instead. This corresponded to a thickness of ∼150 nm (Figure S12). On the other hand, higher concentrations of V1509 negatively affected device performance. The best efficiency of 23% was achieved with a lower concentration of 10 mg/mL, corresponding to a thickness of ∼75 nm. These values are comparable to those of the state-of-the-art spiro-MeOTAD, which exhibited an average PCE of 24%.

The morphology of HTMs under their optimal conditions was compared in Figure S13. The V1508 film exhibited some pinholes and smaller grains in comparison to spiro-MeOTAD, while the V1509 film displayed perovskite grain features owing to its significantly lower thickness. The SEM images, which appeared blurry, indicated the subpar conductivity of all three HTMs.

**Figure 2:**
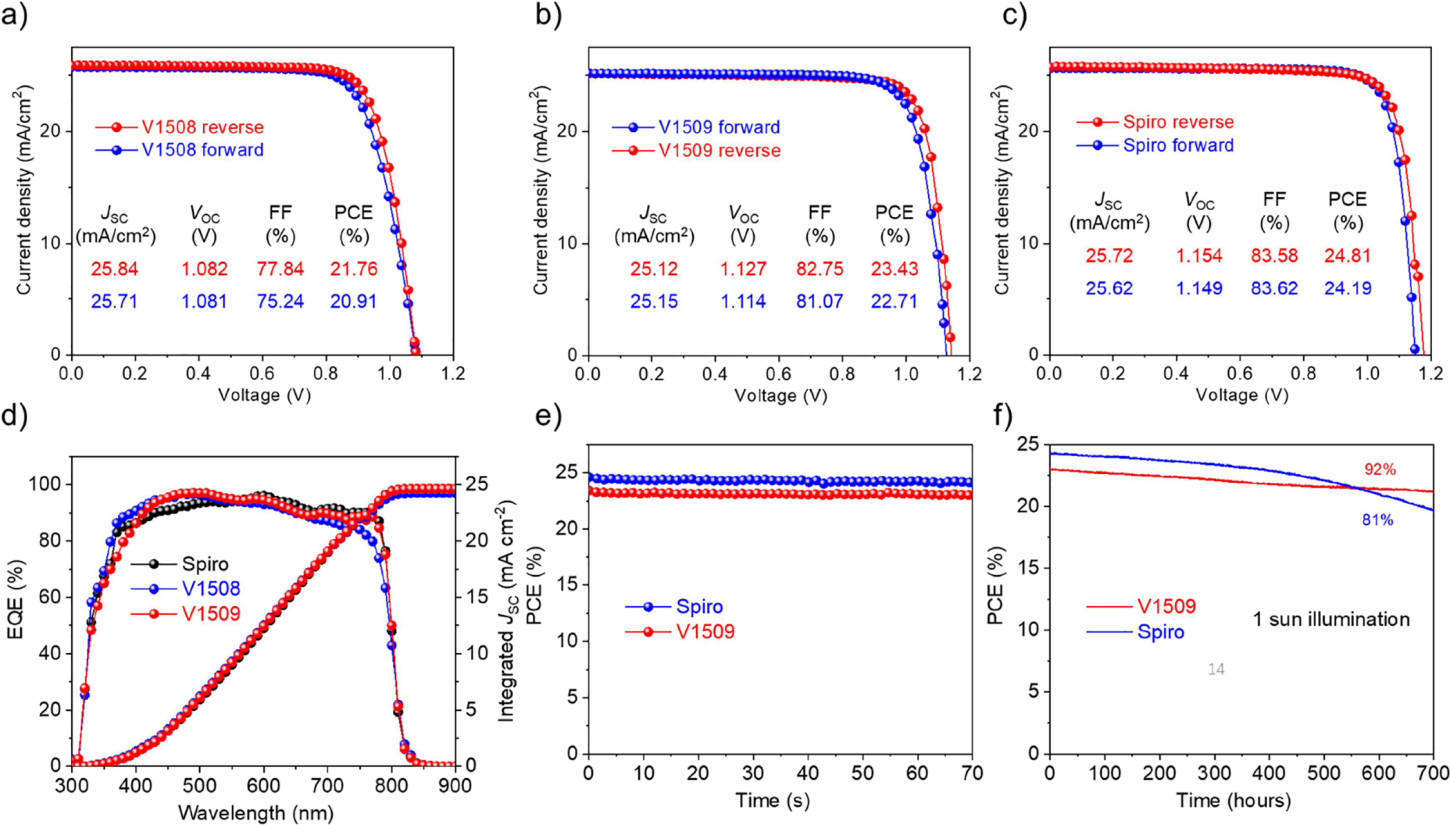
*J*–*V* characteristics of the best-performing (a) V1508-based, (b) V1509-based, and (c) spiro-MeOTAD-based device. (d) EQE spectra of devices. (e) SPO of devices measured at room temperature. (f) PCE evolution of the devices under constant 1 sun illumination.

Figure 2a–c provides the current density–voltage (*J*–*V*) characteristics of the champion PSC devices with their external quantum efficiency (EQE) spectra shown in Figure 2e. The V1509-based device demonstrated a higher PCE of 23.4% compared to the V1508-based device, which had a PCE of 21.8%. This improvement mainly resulted from the higher open-circuit voltage (*V*_oc_) and fill factor, although the short-circuit current (*J*_SC_) was slightly lower. The diminished photovoltaic performance of the V1508-based device can be attributed to the elevated charge transport resistance, diminished charge recombination resistance, and a slower charge extraction process at the perovskite/V1508 interface. This is corroborated by the impedance and transient photocurrent (TPC) results, as illustrated in Figure S14a,b. The photoluminescence quantum yield (PLQY) was used to reveal the nonradiative losses at the perovskite/HTM interface (Figure S14c).^36^ The V1508-based film exhibited a lower PLQY after perovskite contacting with the HTM, indicating a higher nonradiative recombination at the perovskite/V1508 interface and contributing to the lower *V*_oc_. The difference between the V1509 and spiro-MeOTAD-based devices primarily stemmed from the lower hole mobility and lower HOMO level of V1509 compared to spiro-MeOTAD.^34^

To assess the impact of V1509 on device stability, we monitored the steady-state output (SPO) of PSCs under 1 sun illumination (AM 1.5 G) both at room temperature and at 50 °C. As depicted in Figure 2e, both devices showed a stable power output within the measurement duration. When the devices were subjected to a higher temperature of 50 °C, the V1509-based device initially experienced increased PCE followed by stabilization. In contrast, the PCE of the spiro-MeOTAD-based device continuously decreased over time (Figure S15). We evaluated the ambient stability by measuring the device performance of unencapsulated solar cells under approximately 50% relative humidity (RH). After 500 h of aging, the cell based on V1509 HTM showed 9% loss in PCE, while the spiro-MeOTAD-based device exhibited a 18% efficiency loss (Figure S16). The thermal stability was tested on devices at 85 °C (Figure S17). The V1509-based device retained 86% of the initial PCE, while the spiro-MeOTAD-based device only reserved 65% of the initial PCE. We also tracked the device performance at the maximum power point in the ambient with a constant nitrogen flow. As shown in Figure 2f, after 700 h the V1509-based device maintained 92% of its starting efficiency, compared to 81% of the spiro-MeOTAD-based device. Therefore, V1509 HTM demonstrates enhanced long-term humidity and thermal and operational stability compared to spiro-MeOTAD.

## 3 Conclusions

In this study, we developed a straightforward synthetic procedure for the synthesis and application of new hole-transporting materials (HTMs). These HTMs feature various peripheral donors combined with a triphenylethylene central core. We conducted comprehensive investigations into their thermal, optical, photoelectrical, and photovoltaic properties. Notably, these novel HTMs have been successfully utilized in PSCs, showcasing remarkable efficiencies of over 23%. Importantly, the PSC incorporated with the champion HTM V1509 exhibited enhanced long-term stability once compared to PSCs employing spiro-MeOTAD as the p-type semiconductor. An additional advantage of these new HTMs is their lower cost, making them promising candidates for practical applications in the field of solar energy conversion. Overall, this study highlights the potential of the developed HTMs in advancing the performance and stability of PSCs, offering opportunities for more efficient and economically viable solar energy technologies.

## 4 Experimental Section

### 4.1 Materials

Tin(II) chloride dihydrate (99.99%), urea (99%), hydrochloride acid (37 wt % in water), thioglycolic acid (98%), cesium iodide (99.99%), 4-*tert*-butylpyridine (98%), and bis(trifluoromethanesulfonyl)-imide lithium salt (LiTFSI, 99.0%) were purchased from MilliporeSigma. Lead iodide (99.99%) was from TCI America. Formamidinium iodide (FAI), methylammonium chloride (MACl), methylammonium iodide (MAI), phenylethylammonium iodide (PEAI), and FK209 Co(III)TFSI salt were purchased from Greatcell Solar Materials. *N*,*N*-dimethylformamide (DMF, 99.8%), dimethyl sulfoxide (DMSO, 99.7%), isopropanol (99.8%), acetonitrile (99.9%), and chlorobenzene (99.8%) were purchased from the MilliporeSigma. *N*^2^,*N*^2^,*N*^2^′,*N*^2^′,*N*^7^,*N*^7^,*N*^7^′,*N*^7^′-octakis(4-methoxyphenyl)-9,9′-spirobi[9*H*-fluorene]-2,2′,7,7′-tetramine (spiro-OMeTAD) was from the Xi’an polymer light technology corp.

### 4.2 Device Fabrication

The fluorine-doped tin oxide (FTO) glasses were cleaned by ultrasonic under detergent, deionized water, acetone, and isopropyl alcohol each for 15 min. The electron transport layer was prepared by the chemical bath deposition method in a 90 °C oven for 5.5 h. The precursor solution was prepared by mixing 275 mg SnCl_2_·2H_2_O, 1.25 g urea, 1.25 mL HCl, and 25 μL thioglycolic acid in 100 mL of deionized water.^37^ The substrates were annealed at 190 °C for 1 h. Before perovskite deposition, the FTO/SnO_2_ substrates were treated with UV ozone for 30 min. Then, the substrates were transferred to a nitrogen glovebox for perovskite deposition. The perovskite solution (0.07 M CsI, 0.08 M MAI, 1.32 M FAI, 0.5 M MACl, and 1.54 M PbI_2_ in mixed DMF and DMSO solution with a volume ratio of 4:1) was spin-coated on the substrates at 1000 rpm for 10 s and 5000 rpm for 25 s, respectively. At the last 10 s, 200 μL of chlorobenzene was dropped on the films. The perovskite films were annealed at 100 °C for 60 min and 150 °C for 10 min. Twenty mM PEAI isopropanol solution was spin-coated at 5000 rpm for 20 s as the passivation layer. For the solution preparation of hole transport layers (HTLs), 80 mg spiro-OMeTAD was dissolved in 1 mL chlorobenzene, with 32 μL 4-*tert*-butylpyridine, 19 μL LiTFSI solution (517 mg/mL in acetonitrile), and 14 μL FK209 Co(III)TFSI solution (376 mg/mL in acetonitrile). Similarly, new HTMs (V1508-15) were dissolved in chlorobenzene at 10, 40, and 80 mg/mL. For the 80 mg/mL condition, 32 μL of 4-*tert*-butylpyridine, 19 μL of LiTFSI solution, and 14 μL of FK209 Co(III)TFSI solution were added to 1 mL of HTM solution. For the 40 mg/mL condition, 16 μL of 4-*tert*-butylpyridine, 9.5 μL of LiTFSI solution, and 7 μL of FK209 Co(III)TFSI solution were added to 1 mL of HTM solution. For the 10 mg/mL condition, 4 μL 4-*tert*-butylpyridine, 4.75 μL LiTFSI solution, and 3.5 μL FK209 Co(III)TFSI solution were added to 1 mL of HTM solution. The HTLs were deposited at 3000 rpm for 30 s by a spin-coating method. Finally, a 70-nm gold electrode was thermally evaporated under a 10^–7^ Torr vacuum.

### 4.3 Characterizations

The *J*–*V* characteristics were measured in a nitrogen glovebox using a Keithley 2400 source under simulated AM 1.5 G irradiation (100 mW cm^–2^) using a Xe arc lamp from a ScienceTech A1 Light Line Class AAA solar simulator (calibrated by a reference solar cell from Newport before measurement). The scanning step was 20 mV for the solar cells with an active area of 0.0625 cm^2^. The humidity stability tests were conducted in an ambient of 30–40% RH monitored by a hygrometer. Impendence spectroscopy and TPC were conducted using the Fluxim Paios system.

## Supporting Information Available

Detailed synthetic procedures, NMR and MS spectra, DSC, PESA, and SEM data; device statistic, impedance responses, transient photocurrent decays, PLQY and PCE evolution under different atmospheres data (PDF)
