# Supplementary material for: Asymmetric Triphenylethylene-Based Hole Transporting Materials for Highly Efficient Perovskite Solar Cells
Source: ACS Appl Mater Interfaces. 2024 Feb 6;16(6):7310–6. doi: 10.1021/acsami.3c17811 (PMC10875638; doi:10.1021/acsami.3c17811)
Supplement: Supplementary file 1 — am3c17811_si_001.pdf [file am3c17811_si_001.pdf]

Supporting Information

Asymmetric Triphenylethylene-based Hole Transporting Materials for Highly Efficient Perovskite Solar Cells

Julius Petrulevicius,[†a] Yi Yang,[†b] Cheng Liu,[b] Matas Steponaitis,[a] Egidijus Kamarauskas,[d] Maryte Daskeviciene,[a] Abdulaziz S. R. Bati,[b] Tadas Malinauskas,[a] Vygintas Jankauskas,[d] Kasparas Rakstys,[a] Mercouri G. Kanatzidis,[b] Edward H. Sargent,[b,c]* Vytautas Getautis[a]**

[a]Department of Organic Chemistry, Kaunas University of Technology, Radvilenu pl. 19, Kaunas, 50254 Lithuania

[b]Department of Chemistry, Northwestern University, 2145 Sheridan Rd, Evanston, Illinois 60208, United States

[c]Department of Electrical and Computer Engineering, Northwestern University, 2145 Sheridan Rd, Evanston, Illinois 60208, United States

[d]Institute of Chemical Physics Vilnius University, Sauletekio al. 3, Vilnius 10257, Lithuania

†These authors contributed equally to this work.

*Corresponding authors:

m-kanatzidis@northwestern.edu; ted.sargent@northwestern.edu; vytautas.getautis@ktu.lt

***General methods**:* Chemicals required for the synthesis were purchased from Sigma-Aldrich and TCI Europe. All purchased chemicals were used as received without further purification. 4,4',4"-(ethene-1,1,2-triyl)tris(bromobenzene) (**1**) was synthesized as described in the literature[1]. 9-ethyl-*N*-(4-methoxyphenyl)-9*H*-carbazol-3-amine (**2**), 3-bromo-9*H*-fluorene (**3**) and 3-bromo-9,9-dibutyl-9*H*-fluorene (**4**) were synthesized following a previously described procedures[2-4]. Reaction course was monitored by thin-layer chromatography on ALUGRAM SIL G/UV254 plates visualized under UV light. Silica gel (grade 9385, 230–400 mesh, 60 Å, Aldrich) was used for column chromatography. Elemental analysis was performed by an Exeter Analytical CE-440 elemental analyzer, Model 440 C/H/N/. ^{1}H NMR spectra were recorded on a Bruker Avance III spectrometer at 400 MHz with a 5 mm double resonance broad band BBO z-gradient room temperature probe, ^{13}C NMR spectra were recorded using the same instrument at 101 MHz. The chemical shifts (expressed in ppm) are relative to tetramethylsilane (TMS). All NMR experiments were performed at 25 °C. FT-IR spectra ($\bar{\upsilon}$, cm^{-1}) were recorded by using a Perkin–Elmer Frontier spectrophotometer with a single reflectance horizontal ATR (Attenuated Total Reflectance) cell equipped with a diamond crystal. The data were recorded in the spectral range from 560 to 4000 cm^{-1} by accumulating 5 scans with a resolution of 1 cm^{-1}. MS were recorded on Waters SQ Detector 2 Spectrometer using the electrospray ionization (ESI) technique. Thermogravimetric analysis (TGA) was performed on a Q50 thermogravimetric analyzer (TA Instruments) at a scan rate of 10 K/min in a nitrogen atmosphere. Differential scanning calorimetry (DSC) was performed on a Q10 calorimeter (TA Instruments) at a heating rate of 10 K/min in a nitrogen atmosphere. The glass transition temperatures for the investigated compounds were determined during the second heating scan.

***Optical measurements**:* Absorption spectra of the synthesized semiconductors were measured in dilute solutions in Tetrahydrofuran (THF) (concentration 10^{-4} M) and on glass substrate using a

UV/Vis spectrophotometer, Lambda 35 (Perkin–Elmer). The photoluminescence emission spectra were recorded in dilute solutions in THF (concentration 10^{-5} M) and on glass substrate using spectrophotometer, Edinburgh Instruments FLS920.

***Ionization Potential Measurements**:* The ionization potentials were measured by the electron photoemission method in an air atmosphere. The sample solutions in THF were poured onto an aluminum-coated polyester film coated with an adhesive layer of a copolymer of methyl methacrylate and methacrylic acid. A diffraction grating monochromator with a deuterium lamp was used for the experiment. The power of the falling light was ~ $5 \cdot 10^{-8}$ W. A negative voltage (-100 V) was connected to the test sample. The electron photoemission current was measured with an open Geiger–Müller counter[5]. The measurement method error was evaluated as 0.03 eV.

***Hole Drift Mobility Measurements**:* Carrier drift mobility was determined by the time-of-flight (XTOF) method. The sample solutions in THF were poured onto aluminum-coated glass plates. The sample was poured from a solution of pure substance. The layers were dried for 1 hour at 60 °C. The thickness of the transport layer was measured with an optical microscope- interferometer. The drift mobility of electron holes (μ) was determined in electrophotographic mode at an electric field of $(0.1 \div 1) \cdot 10^6$ V/cm. Charge carriers were generated at the layer surface by illumination with nitrogen laser nanosecond pulses (λ = 337 nm).

Molecular design of triphenylethylene based HTMs: While searching for the optimal substituents around the triphenylethylene central core, fluorene derivatives have also been considered. This lead to the synthesis of materials **V1510-V1515** (Scheme S1). Preliminary tests in PSCs were carried out with the new triphenylethylene derivatives: layers were formed from various concentrations of the V series compounds while the dopant concentrations in the solutions remained the same. During these experiments, the devices containing HTMs with carbazole-based substituents (**V1508**, **V1509**) significantly outperformed those containing fluorene moieties (**V1510-V1515**) reaching

over 20% compared to less than 14% efficiency, respectively. Further studies, therefore, were focused on materials **V1508** and **V1509**.

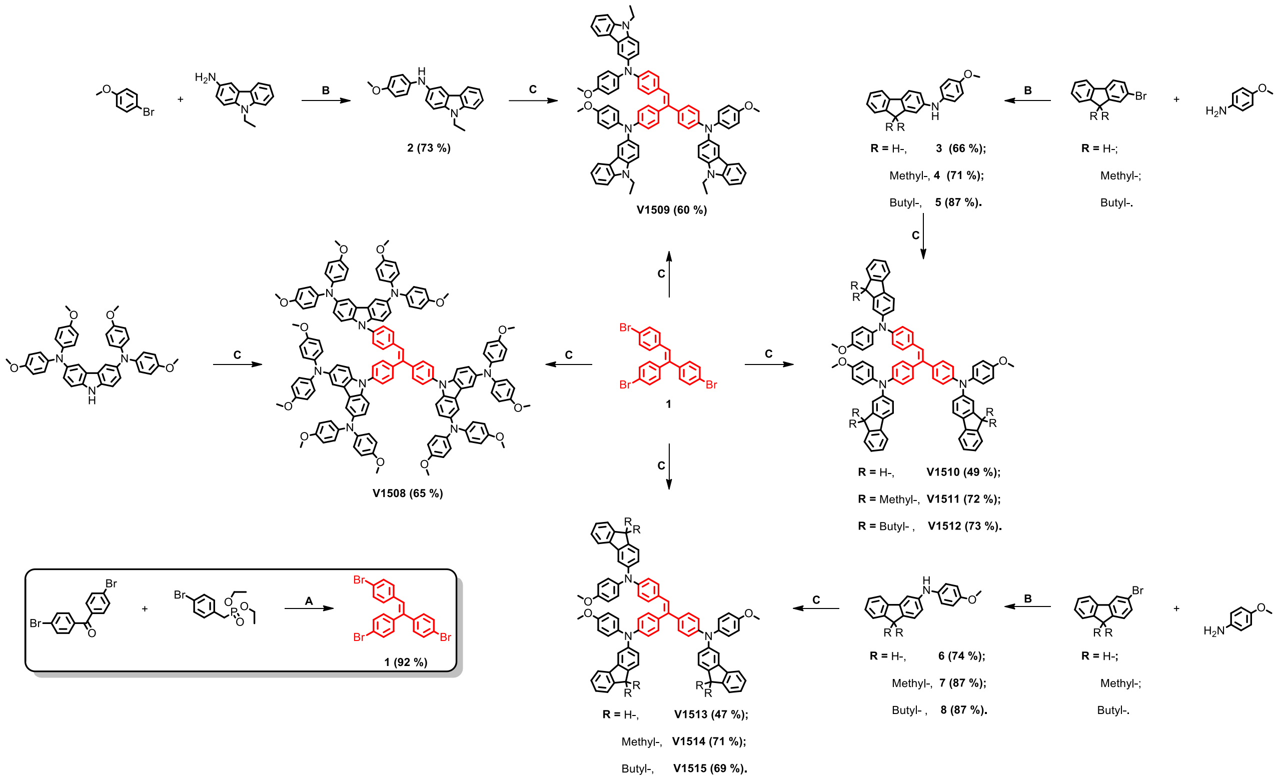

Scheme S1. Synthesis of intermediates **1-8** and HTMs **V1508-V1515**; A – *t*-BuOK, Ar, THF, r. t., 2 h; B – $Pd(OAc)_2$, XPhos, *t*-BuONa, Ar, dioxane, reflux, 45 min; C – $Pd(OAc)_2$, $[(t\text{-}Bu)_3PH]BF_4$, *t*-BuONa, Ar, toluene, reflux, 5 h.

Synthesis:

9-{4-[1,2-bis(4-{3,6-bis[bis(4-methoxyphenyl)amino]-9*H*-carbazol-9-yl}phenyl)ethenyl]phenyl}-*N*^{3},*N*^{3},*N*^{6},*N*^{6}-tetrakis(4-methoxyphenyl)-9*H*-carbazole-3,6-diamine (V1508)

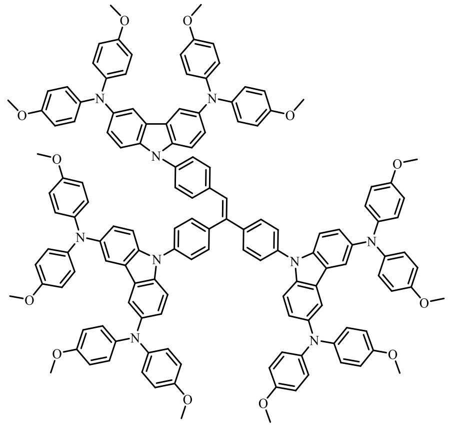

A solution of compound 4,4',4''-(ethene-1,1,2-triyl)tris(bromobenzene) (0.40 g, 0.8 mmol, 1 eq) and *N*^{3},*N*^{3},*N*^{6},*N*^{6}-tetrakis(4-methoxyphenyl)-9*H*-carbazole-3,6-diamine (2.24 g, 3.6 mmol, 4.5 eq) in anhydrous toluene (19.4 mL) was purged with argon for 30 minutes. Afterwards, palladium (II) acetate (0.0074 g, 0.033 mmol, 0.041 eq), tri-*tert*-butylphosphonium tetrafluoroborate (0.0190 g, 0.066 mmol, 0.082 eq) were added, and the mixture was purged with argon for 10 min. Then sodium *tert*-butoxide (0.35 g, 3.6 mmol, 4.5 eq) was added, and the solution was refluxed under an argon atmosphere for 5 hours. After completion of the reaction (TLC, acetone: tetrahydrofuran: *n*-hexane, 5:5:15, v:v:v), the reaction mixture was cooled to room temperature, filtered through celite and poured into acetone (135 ml). The precipitate was filtered off and washed with water and acetone. The crude product was purified by column chromatography (acetone: tetrahydrofuran: *n*-hexane, 4:4:17, v:v:v). The obtained product was dissolved in THF and precipitated into 15 times excess of acetone. The precipitate was filtered off and washed with acetone to collect **V1508** as a

lemon-yellow solid. (0.10 g, 65 %).

<u>^{1}H NMR</u> (400 MHz, THF-d_8) δ: (400 MHz, THF-d_8) δ 7.80 – 7.57 (m, 14H), 7.53 – 7.43 (m, 4H), 7.43 – 7.32 (m, 5H), 7.27 (d, J = 8.7 Hz, 2H), 7.14 – 7.02 (m, 6H), 6.99 – 6.82 (m, 24H), 6.79 – 6.67 (m, 24H), 3.70 (s, 36H).

<u>^{13}C NMR</u> (101 MHz, THF-d_8) δ 156.07, 156.03, 142.79, 142.49, 139.86, 138.38, 137.01, 132.94, 132.00, 129.95, 129.21, 128.10, 127.77, 127.37, 127.03, 125.33, 125.24, 117.47, 115.22, 111.37, 55.58.

Anal. Calcd. for $C_{140}H_{115}N_9O_{12}$: C, 79.49; H, 5.48; N, 5.96; found: C, 79.40; H, 5.42; N, 5.96.

$C_{140}H_{115}N_9O_{12}$ [M$^+$] exact mass = 2113.87, MS (ESI): [M+H$^+$] = 2114.43.

FT-IR, $\bar{\upsilon}$ (cm^{-1}): 3037 (aromatic CH); 2994, 2946, 2930, 2930, 2904, 2829 (aliphatic CH); 1604, 1584, 1500, 1480, 1455, 1440 (C=C); 1233, 1033 (C–O–C).

_N_-{4-[1,2-bis({4-[(9-ethyl-9_H_-carbazol-3-yl)(4-methoxyphenyl)amino]phenyl})ethenyl]phenyl}-9-ethyl-_N_-(4-methoxyphenyl)-9_H_-carbazol-3-amine (V1509)

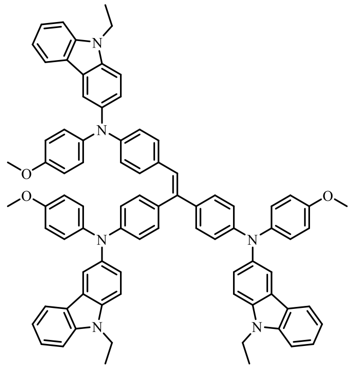

Target compound **V1509** was synthesized from 4,4',4''-(ethene-1,1,2-triyl)tris(bromobenzene) and 9-ethyl-*N*-(4-methoxyphenyl)-9*H*-carbazol-3-amine according the same procedure as

compound **V1508**. After completion of the reaction (TLC, acetone: tetrahydrofuran: *n*-hexane, 4:4:17, v:v:v), the reaction mixture was filtered through celite, extracted with ethyl acetate and distilled water. The organic layer was dried over anhydrous Na_2SO_4, filtered and solvent evaporated. The crude product was purified by column chromatography (acetone: tetrahydrofuran: *n*-hexane, 2:2:21, v:v:v). The obtained product was dissolved in THF and precipitated into 15 times excess ethanol. The precipitate was filtered off and washed with ethanol to collect **V1509** as a lemon-yellow solid. (0.58 g, 60 %).

^{1}H NMR (400 MHz, THF-d_8) δ 7.96 (dd, J = 7.7, 2.8 Hz, 2H), 7.89 (d, J = 6.9 Hz, 4H), 7.49 – 7.31 (m, 9H), 7.24 (dd, J = 11.2, 4.9 Hz, 3H), 7.17 (d, J = 8.7 Hz, 2H), 7.13 – 6.99 (m, 11H), 6.93 (dd, J = 8.5, 3.9 Hz, 4H), 6.89 – 6.72 (m, 11H), 4.39 (dq, J = 14.1, 7.0 Hz, 6H), 3.73 (d, J = 15.8 Hz, 9H), 1.45 – 1.34 (m, 9H).

^{13}C NMR (101 MHz, THF-d_8) δ 156.95, 156.91, 156.89, 149.57, 149.50, 148.69, 142.51, 142.43, 142.39, 141.55, 141.50, 140.79, 140.67, 140.32, 138.09, 136.76, 133.45, 131.63, 131.05, 130.89, 128.55, 127.24, 127.18, 127.01, 126.48, 126.44, 125.99, 125.96, 125.88, 125.85, 124.91, 124.88, 123.77, 123.73, 121.34, 121.26, 120.83, 120.60, 120.19, 119.42, 119.40, 119.16, 119.05, 118.98, 115.36, 110.19, 109.39, 109.35, 55.61, 55.56, 38.14, 14.17, 14.15.

Anal. calcd for $C_{83}H_{70}N_6O_3$: C, 83.11; H, 5.88; N, 7.01; found: C, 83.36; H, 5.98; N, 7.12. $C_{83}H_{70}N_6O_3$ [M^+] exact mass = 1198.55, MS (ESI): [$M+H^+$] = 1199.99.

FT-IR, $\bar{\upsilon}$ (cm^{-1}): 3037 (aromatic CH); 2973, 2931, 2897, 2832 (aliphatic CH); 1600, 1501, 1480, 1460 (C=C); 1239, 1034 (C-O-C).

***N*-{4-[2,2-bis({4-[(9*H*-fluoren-2-yl)(4-methoxyphenyl)amino]phenyl})ethenyl]phenyl}-*N*-(4-methoxyphenyl)-9*H*-fluoren-2-amine (V1510)**

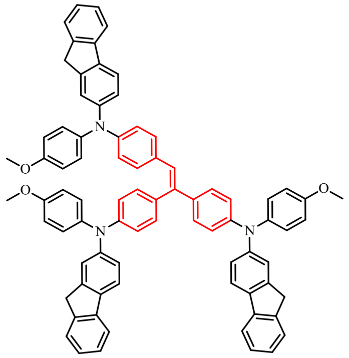

Target triphenylethylene derivative **V1510** was synthesized from 4,4',4"-(ethene-1,1,2-triyl)tris(bromobenzene) and compound **1** according the same procedure as compound **V1508**. After completion of the reaction (TLC, tetrahydrofuran: *n*-hexane, 7:18, v:v), reaction mixture was filtered through celite, extracted with ethyl acetate and distilled water. The organic layer was dried over anhydrous Na_2SO_4, filtered and solvent evaporated. The crude product was purified by column chromatography (tetrahydrofuran: *n*-hexane, 3:22, v:v). The obtained product was dissolved in THF and precipitated into 15 times excess of ethanol. The precipitate was filtered off and washed with ethanol to collect **V1510** as lemon-yellow solid. (0.44 g, 49 %).

<u>^{1}H NMR</u> (400 MHz, THF-d_8) δ 7.73 – 7.57 (m, 6H), 7.48 – 7.43 (m, 2H), 7.41 (d, *J* = 7.5 Hz, 1H), 7.31 – 7.12 (m, 12H), 7.12 – 7.01 (m, 13H), 6.96 (d, *J* = 8.6 Hz, 4H), 6.90 – 6.80 (m, 9H), 3.83 – 3.67 (m, 15H).

<u>^{13}C NMR</u> (101 MHz, THF-d_8) δ 157.67, 157.60, 148.71, 148.63, 148.19, 148.07, 147.79, 145.63, 144.03, 143.99, 142.61, 142.58, 142.55, 141.67, 141.66, 141.57, 140.75, 137.71, 137.45, 137.36, 134.92, 132.21, 131.90, 131.12, 128.73, 128.20, 128.17, 128.13, 127.52, 127.48, 126.63, 125.61, 123.53, 123.41, 123.34, 122.69, 122.18, 121.25, 121.12, 121.03, 120.00, 119.97, 119.94, 115.63, 55.65, 55.61, 37.48.

Anal. calcd for $C_{80}H_{61}N_3O_3$: C, 86.38; H, 5.53; N, 3.78; found: C, 86.13; H, 5.70; N, 3.83. $C_{80}H_{61}N_3O_3$ [M^+] exact mass = 1111.47, MS (ESI): [$M+H^+$] = 1111.90.

FT-IR, $\bar{\upsilon}$ (cm^{-1}): 3036, 3006 (aromatic CH); 2949, 2930, 2904, 2832 (aliphatic CH); 1600, 1574, 1503, 1454 (C=C); 1239, 1034 (C-O-C).

***N*-{4-[2,2-bis({4-[(9,9-dimethyl-9*H*-fluoren-2-yl)(4-methoxyphenyl)amino]phenyl})ethenyl]phenyl}-*N*-(4-methoxyphenyl)-9,9-dimethyl-9*H*-fluoren-2-amine (V1511)**

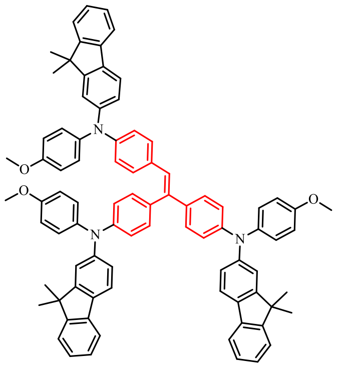

Target compound **V1511** was synthesized from 4,4',4''-(ethene-1,1,2-triyl)tris(bromobenzene) and *N*-(4-methoxyphenyl)-9,9-dimethyl-9*H*-fluoren-2-amine according the same procedure as compound **V1508**. After completion of the reaction (TLC, tetrahydrofuran: *n*-hexane, 1:4, v:v), reaction mixture was filtered through celite, extracted with ethyl acetate and distilled water. The organic layer was dried over anhydrous Na_2SO_4, filtered and solvent evaporated. The crude product was purified by column chromatography (tetrahydrofuran: n-hexane, 2:23, v:v). The obtained product was dissolved in THF and precipitated into 15 times excess of ethanol. The precipitate was filtered off and washed with ethanol to collect **V1511** as lemon-yellow solid. (0.69 g, 72 %).

^{1}H NMR (400 MHz, THF-d_8) δ 7.67 – 7.62 (m, 2H), 7.59 (dd, *J* = 8.2, 2.6 Hz, 3H), 7.54 (d, *J* = 8.2 Hz, 1H), 7.39 (d, *J* = 7.3 Hz, 2H), 7.32 – 7.14 (m, 12H), 7.14 – 7.02 (m, 10H), 7.02 – 6.95 (m, 7H), 6.92 – 6.80 (m, 9H), 3.77 (s, 6H), 3.74 (s, 3H), 1.39 (d, *J* = 6.0 Hz, 12H), 1.29 (s, 6H).

^{13}C NMR (101 MHz, THF-d_8) 157.68, 157.66, 157.56, 155.92, 155.88, 154.43, 154.42, 154.34, 148.70, 148.66, 148.58, 148.54, 148.47, 147.79, 141.61, 141.56, 141.51, 140.70, 140.08, 140.07, 140.03, 137.72, 135.09, 134.82, 134.70, 134.60, 132.26, 131.92, 131.10, 128.73, 128.15, 128.11, 127.92, 127.77, 127.75, 127.71, 127.18, 127.14, 127.09, 126.61, 123.68, 123.59, 123.32, 123.23, 123.20, 122.67, 122.23, 121.40, 121.38, 121.34, 120.10, 120.06, 120.02, 118.80, 118.49, 115.62, 115.59, 55.64, 55.60, 47.54, 47.44, 27.48, 27.44.

Anal. calcd for $C_{86}H_{73}N_3O_3$: C, 86.33; H, 6.15; N, 3.51; found: C, 86.50; H, 6.12; N, 3.75. $C_{86}H_{73}N_3O_3$ [M$^+$] exact mass = 1195.57, MS (ESI): [M+H$^+$] = 1196.06.

FT-IR, $\bar{\upsilon}$ (cm^{-1}): 3036, 3007 (aromatic CH); 2956, 2924, 2902, 2859, 2833 (aliphatic CH); 1600, 1503, 1448 (C=C); 1238, 1035 (C-O-C).

***N*-{4-[2,2-bis({4-[(9,9-dibutyl-9*H*-fluoren-2-yl)(4-methoxyphenyl)amino]phenyl})ethenyl]phenyl}-9,9-dibutyl-*N*-(4-methoxyphenyl)-9*H*-fluoren-2-amine (V1512)**

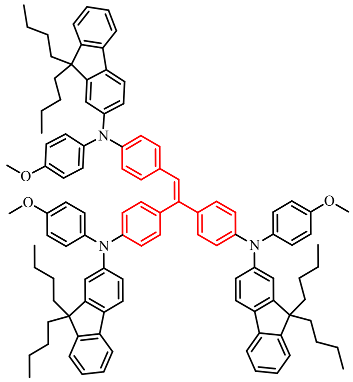

Target compound **V1512** was synthesized from 4,4',4''-(ethene-1,1,2-triyl)tris(bromobenzene) and compound **2** according the same procedure as compound **V1508**. After completion of the reaction (TLC, tetrahydrofuran: *n*-hexane, 3:22, v:v), reaction mixture was filtered through celite, extracted with ethyl acetate and distilled water. The organic layer was dried over anhydrous Na_2SO_4, filtered

and solvent evaporated. The crude product was purified by column chromatography (tetrahydrofuran: *n*-hexane, 1:24, v:v). The obtained product was dissolved in THF and precipitated into 15 times excess of ethanol. The precipitate was filtered off and washed with ethanol to collect **V1512** as lemon-yellow solid. (0.85 g, 73 %).

^{1}H NMR (400 MHz, THF-d_8) δ 7.67 – 7.49 (m, 6H), 7.33 – 7.13 (m, 13H), 7.13 – 7.03 (m, 11H), 7.02 – 6.94 (m, 7H), 6.91 – 6.80 (m, 9H), 3.78 (s, 3H), 3.77 (s, 3H), 3.74 (s, 3H), 2.00 – 1.76 (m, 12H), 1.15 – 0.96 (m, 12H), 0.76 – 0.47 (m, 30H).

^{13}C NMR (101 MHz, THF-d_8) δ 157.60, 157.51, 157.44, 152.78, 152.75, 152.70, 151.32, 151.30, 148.65, 148.57, 148.44, 148.42, 148.23, 147.69, 142.14, 142.11, 141.68, 141.63, 141.53, 140.54, 137.75, 137.03, 136.96, 134.94, 132.10, 131.79, 131.03, 128.65, 127.97, 127.70, 127.64, 127.02, 126.55, 123.55, 123.44, 123.36, 122.64, 121.99, 121.15, 121.12, 119.88, 119.85, 119.40, 119.38, 119.31, 115.58, 115.52, 55.74, 55.72, 55.64, 55.61, 40.94, 40.87, 40.85, 27.03, 27.01, 23.96, 23.94, 14.37, 14.33, 14.32.

Anal. calcd for $C_{104}H_{109}N_3O_3$: C, 86.21; H, 7.58; N, 2.90; found: C, 86.39; H, 7.58; N, 2.99.

$C_{104}H_{109}N_3O_3$ [M$^+$] exact mass = 1447.85, MS (ESI): [M+H$^+$] = 1448.42.

FT-IR, $\bar{\upsilon}$ (cm^{-1}): 3036, 3007 (aromatic CH); 2954, 2928, 2858 (aliphatic CH); 1601, 1504, 1451 (C=C); 1240, 1037 (C-O-C).

***N*-{4-[1,2-bis({4-[(9*H*-fluoren-3-yl)(4-methoxyphenyl)amino]phenyl})ethenyl]phenyl}-*N*-(4-methoxyphenyl)-9*H*-fluoren-3-amine (V1513)**

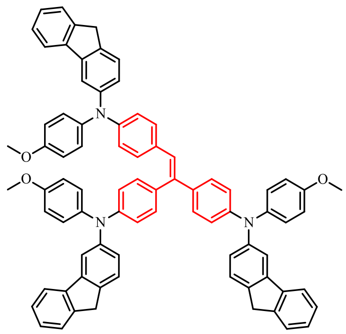

Target compound **V1513** was synthesized from 4,4',4''-(ethene-1,1,2-triyl)tris(bromobenzene) and compound **5** according to the same procedure as compound **V1508**. After completion of the reaction (TLC, tetrahydrofuran: *n*-hexane, 7:18, v:v), reaction mixture was filtered through celite, extracted with ethyl acetate and distilled water. The organic layer was dried over anhydrous Na_2SO_4, filtered and solvent evaporated. The crude product was purified by column chromatography (tetrahydrofuran: *n*-hexane, 3:22, v:v). The obtained product was dissolved in THF and precipitated into 15 times excess of ethanol. The precipitate was filtered off and washed with ethanol to collect **V1513** as lemon-yellow solid. (0.42 g, 47 %).

^{1}H NMR (400 MHz, THF-d_8) δ 7.73 – 7.61 (m, 2H), 7.60 – 7.53 (m, 4H), 7.52 – 7.44 (m, 3H), 7.40 (d, J = 8.1 Hz, 2H), 7.34 (d, J = 8.1 Hz, 1H), 7.30 – 7.14 (m, 8H), 7.13 – 7.05 (m, 8H), 7.04 – 6.92 (m, 9H), 6.90 – 6.78 (m, 9H), 3.84 (s, 4H), 3.80 (s, 2H), 3.76 (s, 6H), 3.73 (s, 3H).

^{13}C NMR (101 MHz, THF-d_8) δ 157.51, 157.50, 157.44, 148.89, 148.82, 148.25, 148.22, 148.14, 148.01, 144.86, 144.82, 144.00, 143.95, 142.49, 142.48, 142.42, 141.84, 141.79, 141.73, 140.62, 138.73, 138.64, 137.53, 134.59, 131.94, 131.83, 131.08, 128.70, 127.95, 127.90, 127.55, 127.51, 127.45, 127.43, 126.50, 126.39, 125.69, 125.65, 124.38, 124.23, 122.74, 122.15, 121.69, 120.84, 120.83, 120.69, 116.75, 116.63, 116.57, 115.57, 55.63, 55.59, 37.05, 37.02.

Anal. calcd for $C_{80}H_{61}N_3O_3$: C, 86.38; H, 5.53; N, 3.78; found: C, 86.30; H, 5.63; N, 3.76. $C_{80}H_{61}N_3O_3$ [M^+] exact mass = 1111.47, MS (ESI): [$M+H^+$] = 1111.91.

FT-IR, $\bar{\upsilon}$ (cm^{-1}): 3038, 3014 (aromatic CH); 2950, 2930, 2902, 2832 (aliphatic CH); 1600, 1502, 1450 (C=C); 1238, 1034 (C-O-C).

***N*-{4-[1,2-bis({4-[(9,9-dimethyl-9*H*-fluoren-3-yl)(4-methoxyphenyl)amino]phenyl})ethenyl]phenyl}-*N*-(4-methoxyphenyl)-9,9-dimethyl-9*H*-fluoren-3-amine (V1514)**

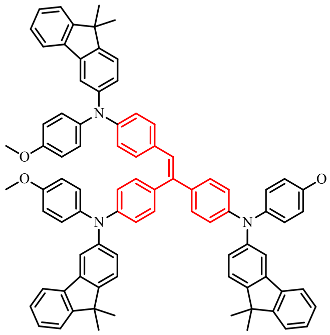

A target compound **V1514** was synthesized from 4,4',4''-(ethene-1,1,2-triyl)tris(bromobenzene) and compound **6** according to the same procedure as compound **V1508**. After completion of the reaction (TLC, tetrahydrofuran: *n*-hexane, 1:4, v:v), reaction mixture was filtered through celite, extracted with ethyl acetate and distilled water. The organic layer was dried over anhydrous Na_2SO_4, filtered and solvent evaporated. The crude product was purified by column chromatography (tetrahydrofuran:*n*-hexane, 2:23, v:v). The obtained product was dissolved in THF and precipitated into 15 times excess of ethanol. The precipitate was filtered off and washed with ethanol to collect **V1514** as lemon-yellow solid. (0.68 g, 71 %).

^{1}H NMR (400 MHz, THF-*d*$_8$) δ 7.63 – 7.55 (m, 2H), 7.53 – 7.46 (m, 4H), 7.45 – 7.37 (m, 3H), 7.33 (d, *J* = 8.1 Hz, 2H), 7.29 (d, *J* = 8.1 Hz, 1H), 7.27 – 7.16 (m, 7H), 7.15 – 7.05 (m, *J* = 16.3, 8.0 Hz, 9H), 7.05 – 6.91 (m, 9H), 6.91 – 6.78 (m, 9H), 3.76 (s, 6H), 3.73 (s, 3H), 1.46 (s, 12H), 1.44 (s,

6H).

<u>^{13}C NMR</u> (101 MHz, THF-d_8) δ 157.56, 157.51, 157.47, 155.03, 154.98, 148.99, 148.96, 148.89, 148.83, 148.36, 148.33, 148.23, 147.99, 141.79, 141.74, 141.68, 141.44, 141.41, 140.62, 139.95, 139.89, 137.56, 134.60, 131.96, 131.83, 131.07, 128.70, 128.10, 128.06, 127.97, 127.94, 127.70, 127.67, 127.65, 126.49, 124.50, 124.48, 123.97, 123.29, 123.25, 122.78, 122.21, 121.74, 120.96, 120.94, 120.81, 116.65, 116.63, 116.57, 115.58, 55.63, 55.59, 47.15, 47.12, 27.59.

Anal. calcd for $C_{86}H_{73}N_3O_3$: C, 86.33; H, 6.15; N, 3.51; found: C, 86.39; H, 6.20; N, 3.57. $C_{86}H_{73}N_3O_3$ [M$^+$] exact mass = 1195.57, MS (ESI): [M+H$^+$] = 1196.06.

FT-IR, $\bar{\upsilon}$ (cm^{-1}): 3039, 3004 (aromatic CH); 2956, 2926, 2902, 2860, 2833 (aliphatic CH); 1601, 1503, 1448 (C=C); 1238, 1035 (C-O-C).

***N*-{4-[1,2-bis({4-[(9,9-dibutyl-9*H*-fluoren-3-yl)(4-methoxyphenyl)amino]phenyl})ethenyl]phenyl}-9,9-dibutyl-*N*-(4-methoxyphenyl)-9*H*-fluoren-3-amine (V1515)**

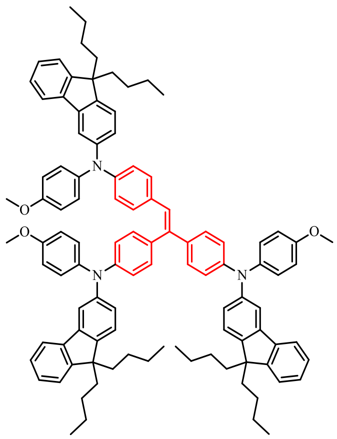

A target compound **V1515** was synthesized from 4,4',4''-(ethene-1,1,2-triyl)tris(bromobenzene) and compound **7** according to the same procedure as compound **V1508**. After completion of the reaction (TLC, tetrahydrofuran: *n*-hexane, 3:22, v:v), reaction mixture was filtered through celite, extracted with ethyl acetate and distilled water. The organic layer was dried over anhydrous Na_2SO_4,

filtered and solvent evaporated. The crude product was purified by column chromatography (tetrahydrofuran:*n*-hexane, 0.1:4.9, v:v). The obtained product was dissolved in THF and precipitated into 15 times excess of ethanol. The precipitate was filtered off and washed with ethanol to collect **V1515** as lemon-yellow solid. (0.80 g, 69 %).

^{1}H NMR (400 MHz, THF-d_8) δ 7.60 – 7.54 (m, 2H), 7.52 – 7.44 (m, 4H), 7.37 – 7.29 (m, 3H), 7.27 – 7.16 (m, 10H), 7.15 – 7.06 (m, 9H), 7.05 – 6.93 (m, 9H), 6.92 – 6.81 (m, 9H), 3.77 (s, 6H), 3.74 (s, 3H), 2.05 – 1.92 (m, 12H), 1.17 – 1.04 (m, 12H), 0.79 – 0.59 (m, 30H).

^{13}C NMR (101 MHz, THF-d_8) δ 157.65, 157.57, 151.98, 151.92, 148.86, 148.78, 148.13, 148.05, 147.98, 147.89, 145.66, 145.60, 145.53, 143.28, 143.26, 141.98, 141.95, 141.91, 141.73, 141.67, 141.63, 140.65, 137.65, 134.57, 132.07, 131.83, 131.10, 128.71, 128.31, 128.21, 128.13, 127.90, 127.88, 127.55, 127.49, 127.47, 126.57, 124.14, 123.96, 123.76, 123.56, 123.52, 122.79, 122.45, 121.95, 120.71, 120.70, 120.58, 115.99, 115.74, 115.64, 55.63, 55.59, 55.31, 41.05, 27.05, 24.03, 14.27, 14.26.

Anal. calcd for $C_{104}H_{109}N_3O_3$: C, 86.21; H, 7.58; N, 2.90; found: C, 86.27; H, 7.60; N, 2.95. $C_{104}H_{109}N_3O_3$ [M^+] exact mass = 1447,85, MS (ESI): [$M+H^+$] = 1448.41. FT-IR, ῡ (cm^{-1}): 3040, 3003 (aromatic CH); 2954, 2928, 2858 (aliphatic CH); 1601, 1504, 1449 (C=C); 1239, 1036 (C-O-C).

***N*-(4-methoxyphenyl)-9*H*-fluoren-3-amine (5)**

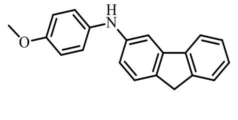

Compound **5** was synthesized from *p*-anisidine and compound **3** according to the same procedure as compound **1**. After completion of the reaction (TLC, acetone:*n*-hexane, 1:4, v:v), reaction mixture was cooled to room temperature, extracted with ethyl acetate and distilled water. The organic layer was dried over anhydrous Na_2SO_4, filtered and the solvent evaporated. The crude

product was purified by column chromatography using 0.1:9 v:v acetone: *n*-hexane as an eluent. Pale yellow solid was collected as a final product. (1.07 g, 74 %).

<u>^{1}H NMR</u> (400 MHz, THF-*d*$_8$) δ 7.66 (d, *J* = 7.5 Hz, 1H), 7.47 (d, *J* = 7.3 Hz, 1H), 7.40 (s, 1H), 7.33 – 7.24 (m, 2H), 7.24 – 7.18 (m, 1H), 7.08 (d, *J* = 8.6 Hz, 2H), 7.01 (s, 1H), 6.88 (d, *J* = 8.1 Hz, 1H), 6.84 (d, *J* = 8.6 Hz, 2H), 3.77 (s, 2H), 3.74 (s, 3H).

<u>^{13}C NMR</u> (101 MHz, THF-*d*$_8$) δ 155.70, 145.93, 145.14, 143.52, 143.03, 138.16, 134.62, 127.26, 127.17, 126.06, 125.67, 121.58, 120.32, 116.22, 115.27, 107.60, 55.63, 36.82.

***N*-(4-methoxyphenyl)-9,9-dimethyl-9*H*-fluoren-3-amine (6)**

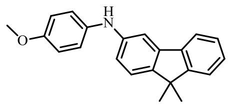

Compound **6** was synthesized from *p*-anisidine and 3-bromo-9,9-dimethyl-9*H*-fluorene according to the same procedure as compound **1**. After completion of the reaction (TLC, acetone: *n*-hexane, 1:4, v:v), reaction mixture was cooled to room temperature, extracted with ethyl acetate and distilled water. The organic layer was dried over anhydrous Na_2SO_4, filtered and the solvent evaporated. The crude product was purified by column chromatography using 0.3:4.7 v:v acetone : *n*-hexane as an eluent. Pale brown solid was collected as a final product (1.38, 87 %).

<u>^{1}H NMR</u> (400 MHz, THF-*d*$_8$) δ 7.64 – 7.56 (m, 1H), 7.45 – 7.36 (m, 1H), 7.32 (s, 1H), 7.28 – 7.19 (m, 3H), 7.08 (d, *J* = 8.7 Hz, 2H), 7.01 (s, 1H), 6.87 (d, *J* = 8.6 Hz, 1H), 6.83 (d, *J* = 8.7 Hz, 2H), 3.74 (s, 3H), 1.43 (s, 6H).

<u>^{13}C NMR</u> (101 MHz, THF-*d*$_8$) 155.72, 155.33, 146.12, 145.19, 140.96, 140.53, 138.16, 127.72, 127.49, 123.64, 123.24, 121.67, 120.44, 116.37, 115.26, 107.72, 55.63, 46.86, 27.77.

9,9-dibutyl-*N*-(4-methoxyphenyl)-9*H*-fluoren-3-amine (7)

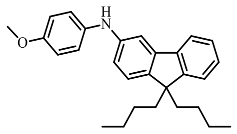

Compound **7** was synthesized from *p*-anisidine and compound **4** according to the same procedure as compound **1**. After completion of the reaction (TLC, tetrahydrofuran: *n*-hexane, 1:4, v:v), reaction mixture was cooled to room temperature, extracted with ethyl acetate and distilled water. The organic layer was dried over anhydrous Na_2SO_4, filtered and the solvent evaporated. The crude product was purified by column chromatography using 1:24 v:v acetone: *n*-hexane as an eluent. Pale brown resin was collected as a final product (1.74 g, 87 %).

^{1}H NMR (400 MHz, THF-*d*$_8$) δ 7.61 – 7.55 (m, 1H), 7.34 – 7.28 (m, 2H), 7.25 – 7.19 (m, 2H), 7.15 – 7.07 (m, 3H), 7.02 (s, 1H), 6.90 – 6.81 (m, 3H), 3.75 (s, 3H), 2.01 – 1.91 (m, 4H), 1.15 – 1.03 (m, 4H), 0.76 – 0.54 (m, 10H).

^{13}C NMR (101 MHz, THF-*d*$_8$) δ 155.77, 152.24, 146.02, 142.95, 142.55, 141.61, 137.96, 127.55, 127.33, 123.90, 123.42, 121.90, 120.19, 115.96, 115.23, 107.18, 55.62, 55.03, 41.28, 27.02, 24.06, 14.27.

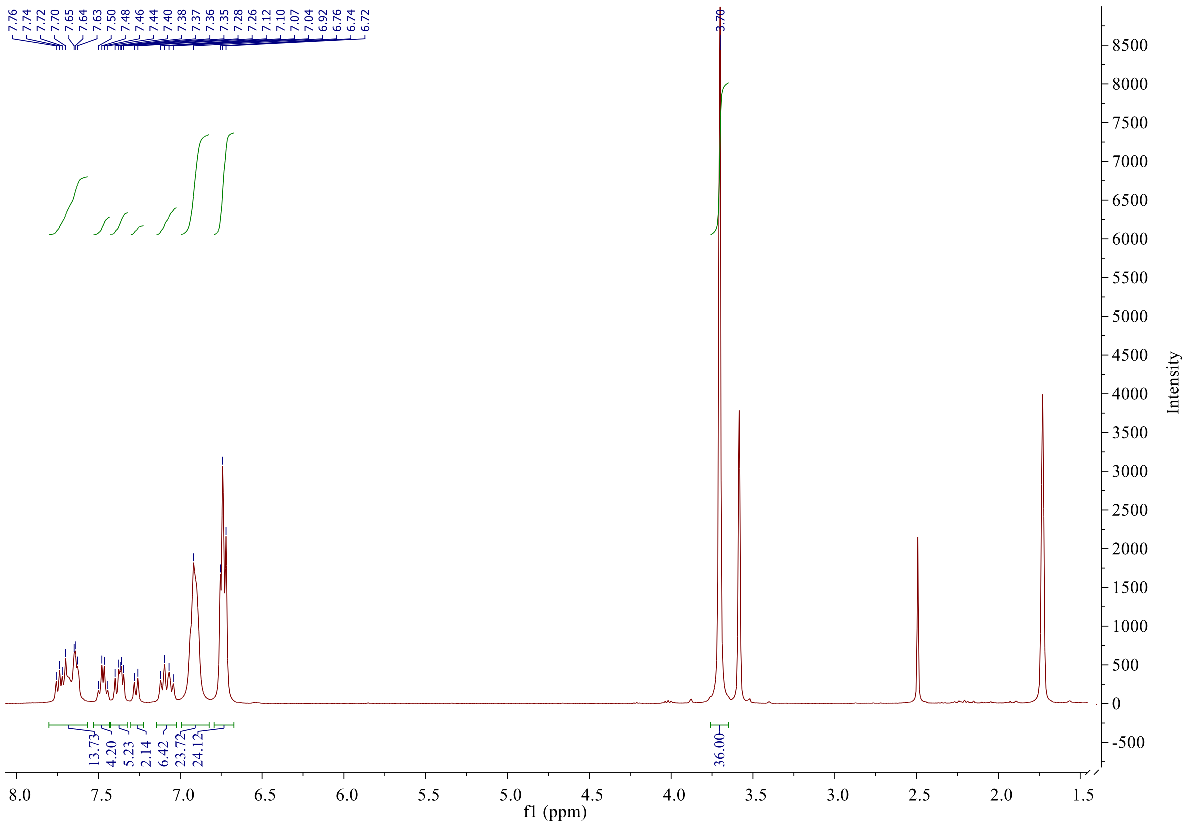
7.76
7.74
7.72
7.70
7.65
7.64
7.63
7.50
7.48
7.46
7.44
7.40
7.38
7.37
7.36
7.35
7.28
7.26
7.12
7.10
7.07
7.04
6.92
6.76
6.74
6.72
3.70
13.73
4.20
5.23
2.14
6.42
23.72
24.12
36.00
f1 (ppm)
Intensity

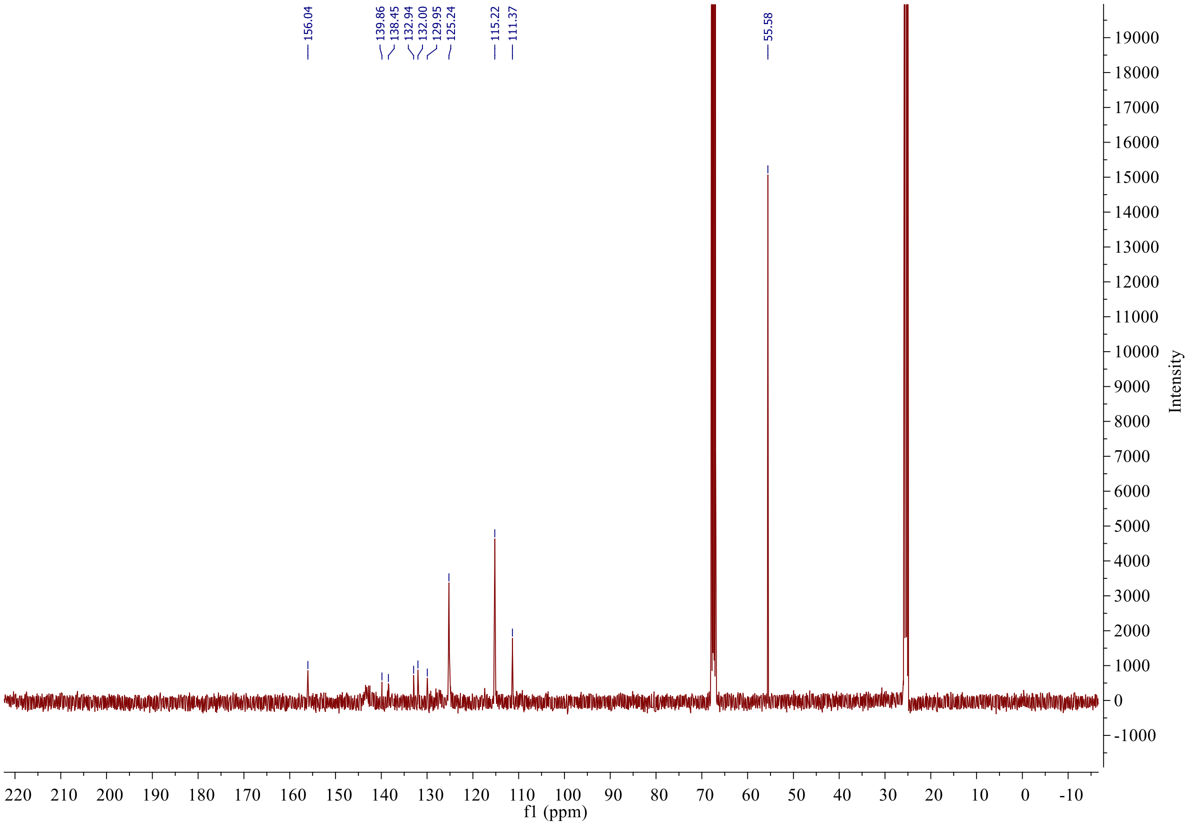
156.04
139.86
138.45
132.94
132.00
129.95
125.24
115.22
111.37
55.58
f1 (ppm)
Intensity

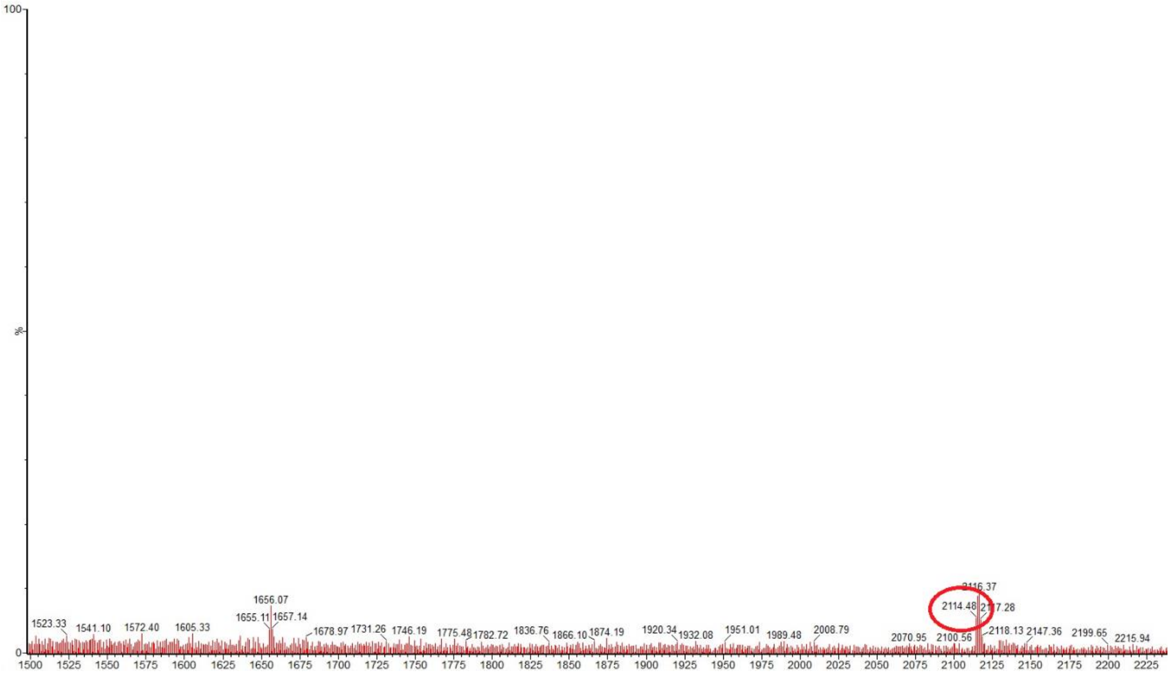

Figure S1. ^{1}H, ^{13}C NMRs and MS spectra of **V1508**

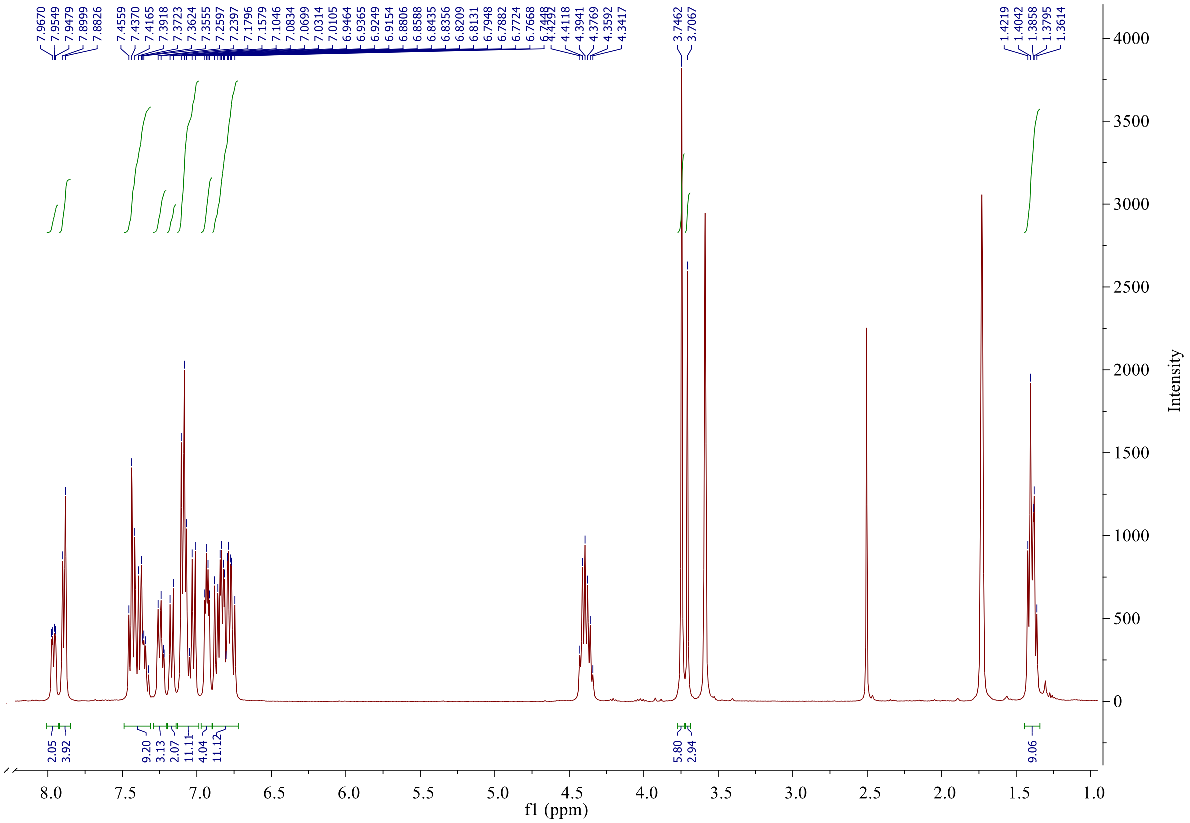
7.9670
7.9549
7.9479
7.8999
7.8826
7.4559
7.4370
7.4165
7.3918
7.3723
7.3624
7.3555
7.2597
7.2397
7.1796
7.1579
7.1046
7.0834
7.0699
7.0314
7.0105
6.9464
6.9365
6.9249
6.9154
6.8806
6.8588
6.8435
6.8356
6.8209
6.8131
6.7948
6.7882
6.7724
6.7668
4.4118
4.3941
4.3769
4.3592
4.3417
3.7462
3.7067
1.4219
1.4042
1.3858
1.3795
1.3614
2.05
3.92
9.20
3.13
2.07
11.11
4.04
11.12
5.80
2.94
9.06
f1 (ppm)
Intensity

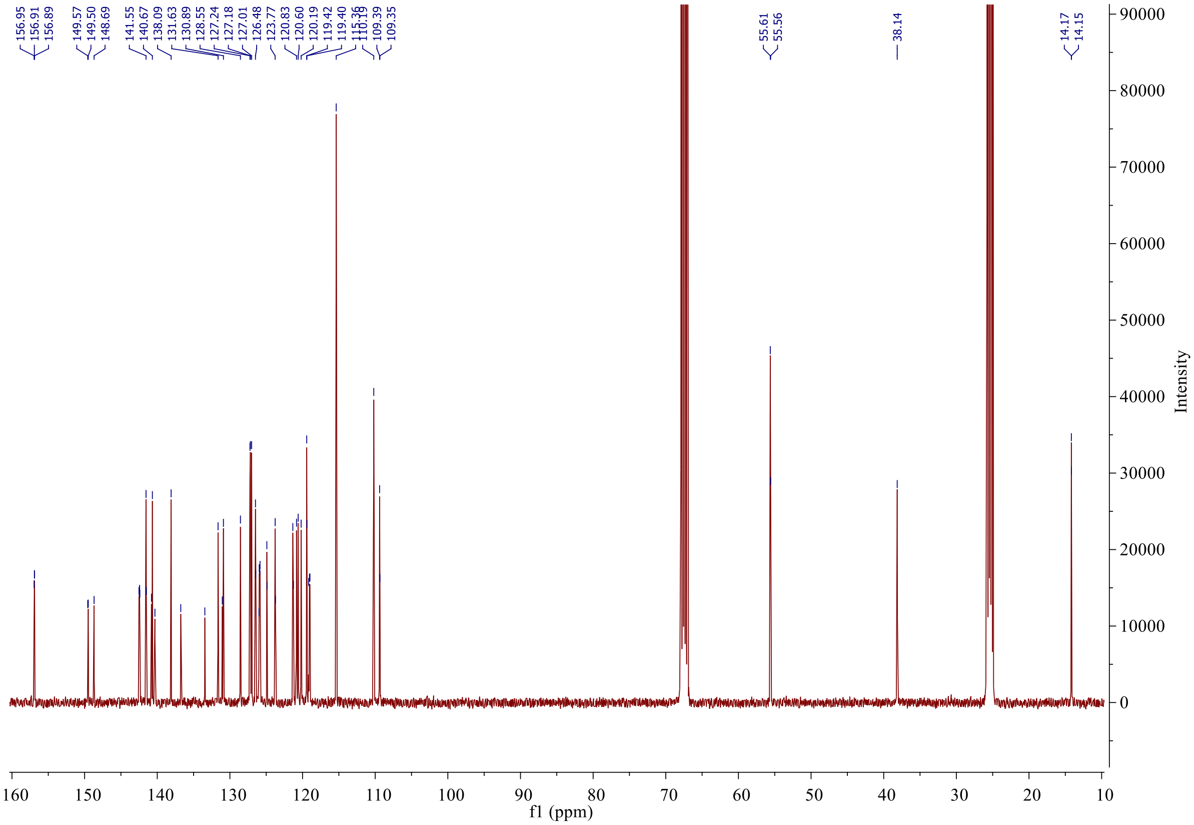
156.95
156.91
156.89
149.57
149.50
148.69
141.55
140.67
138.09
131.63
130.89
128.55
127.24
127.18
127.01
126.48
123.77
120.83
120.60
120.19
119.42
119.40
109.39
109.35
55.61
55.56
38.14
14.17
14.15
f1 (ppm)
Intensity

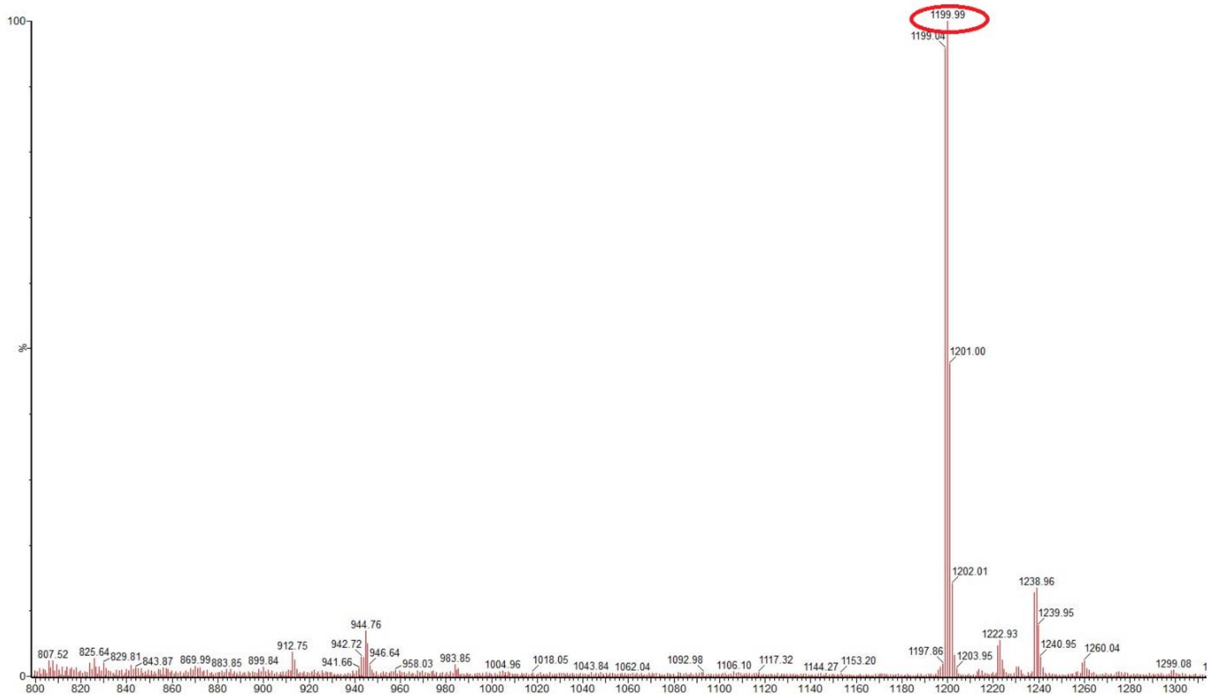

Figure S2. ^{1}H, ^{13}C NMRs and MS spectra of **V1509**

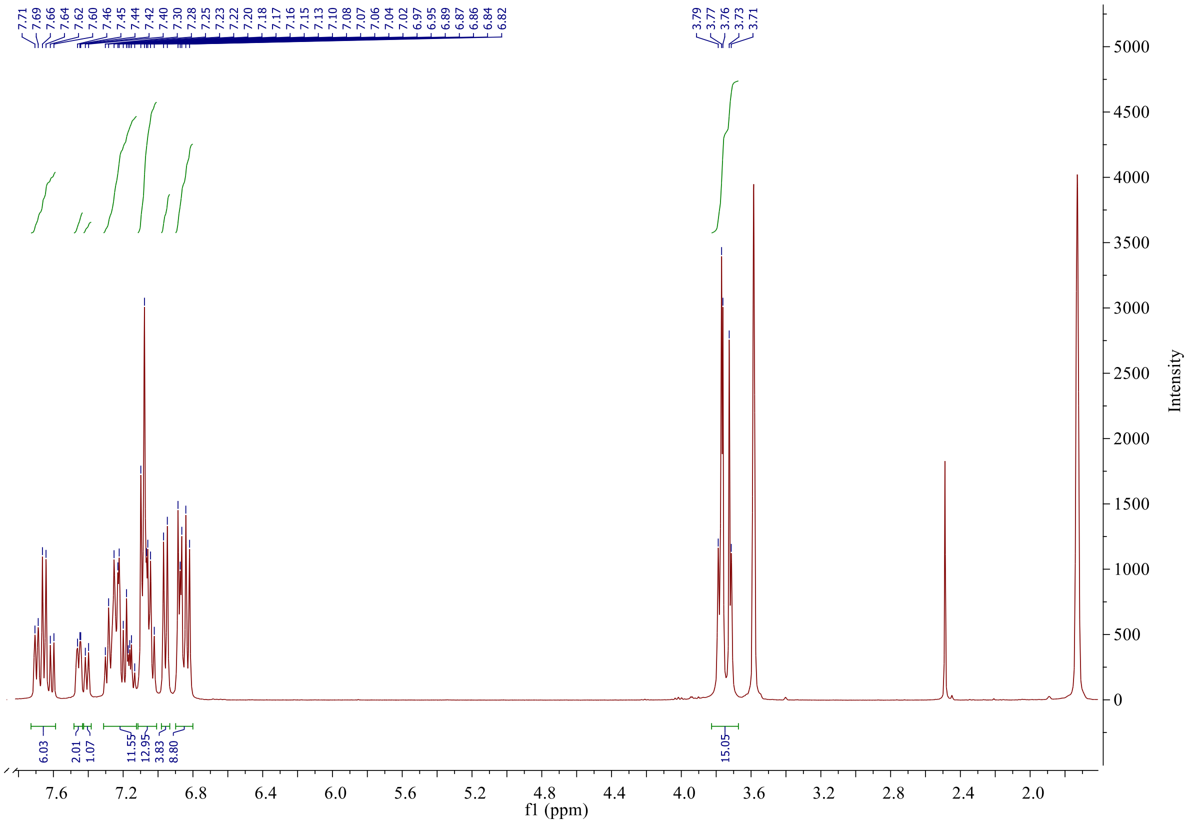

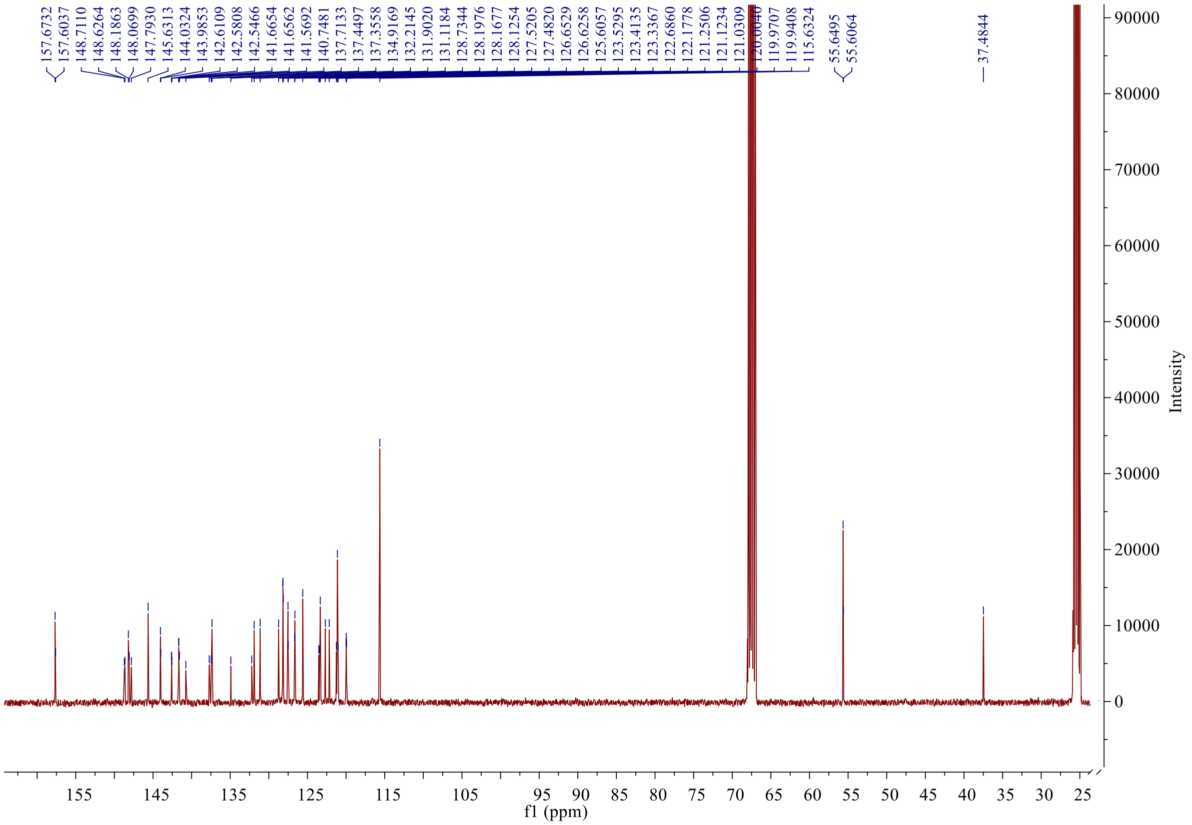

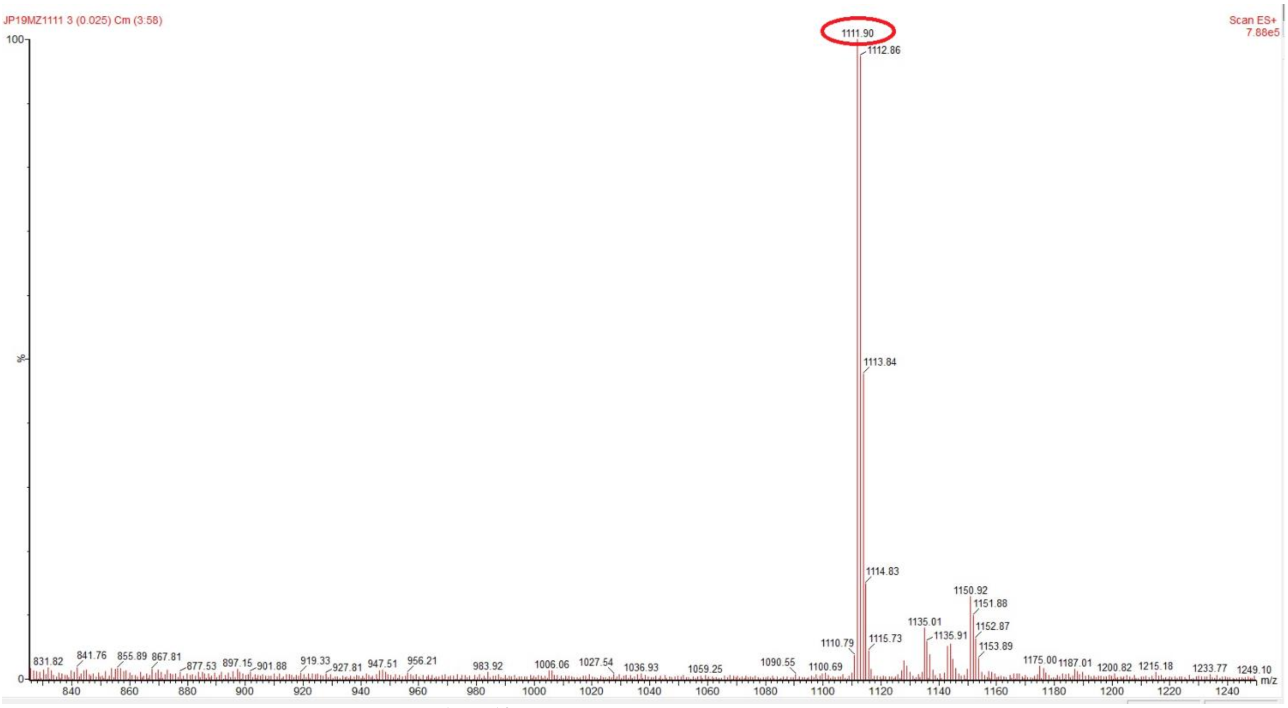

Figure S3. ^{1}H, ^{13}C NMRs and MS spectra of **V1510**

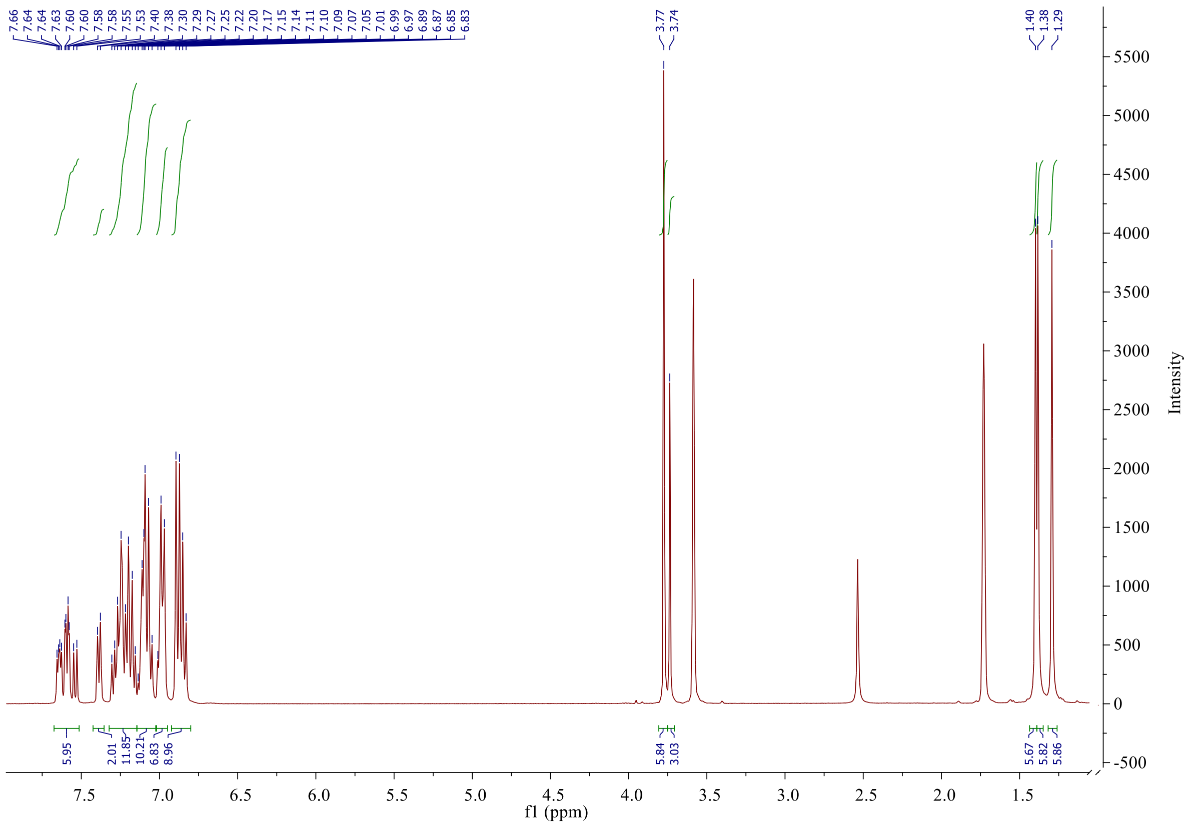
7.66
7.64
7.64
7.63
7.60
7.60
7.58
7.58
7.55
7.53
7.40
7.38
7.30
7.29
7.27
7.25
7.22
7.20
7.17
7.15
7.14
7.11
7.10
7.09
7.07
7.05
7.01
6.99
6.97
6.89
6.87
6.85
6.83
3.77
3.74
1.40
1.38
1.29
5.95
2.01
11.85
10.21
6.83
8.96
5.84
3.03
5.67
5.82
5.86
f1 (ppm)
Intensity

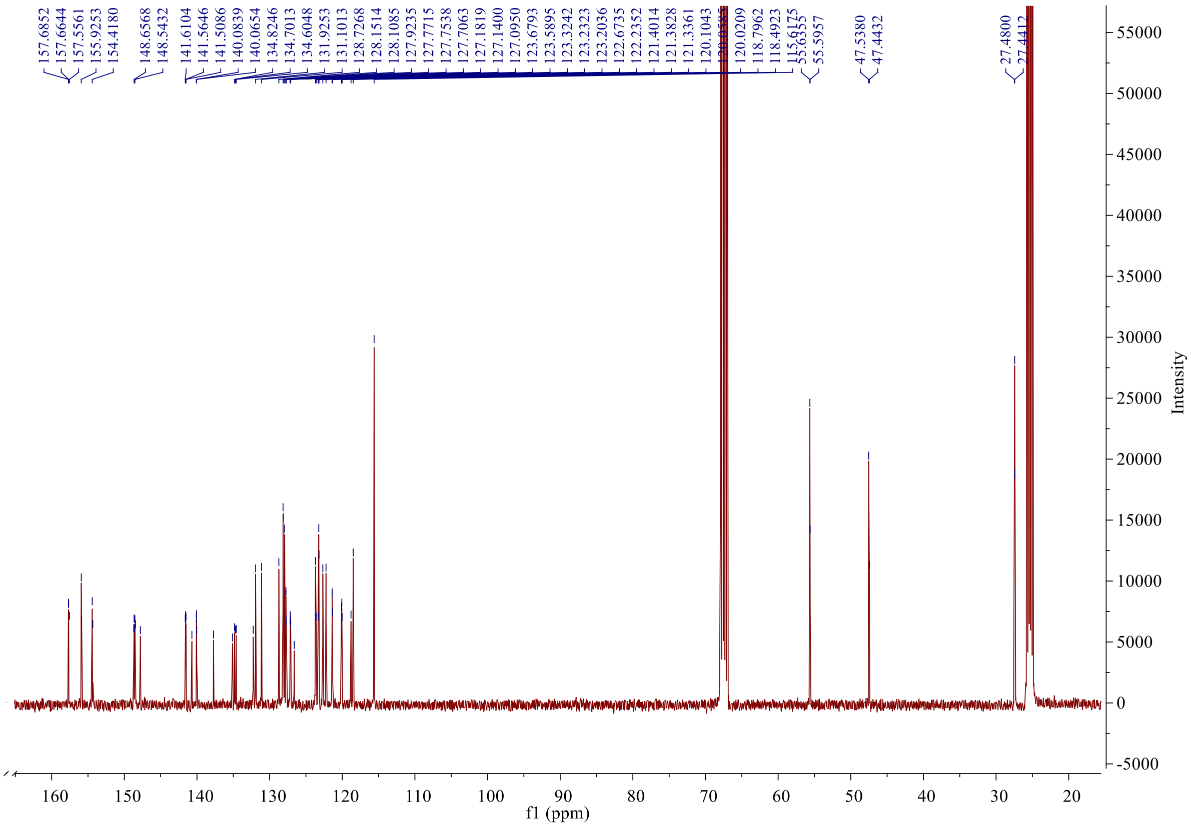
157.6852
157.6644
157.5561
155.9253
154.4180
148.6568
148.5432
141.6104
141.5646
141.5086
140.0839
140.0654
134.8246
134.7013
134.6048
131.9253
131.1013
128.7268
128.1514
128.1085
127.9235
127.7715
127.7538
127.7063
127.1819
127.1400
127.0950
123.6793
123.5895
123.3242
123.2323
123.2036
122.6735
122.2352
121.4014
121.3828
121.3361
120.1043
120.0209
118.7962
118.4923
115.6175
55.6355
55.5957
47.5380
47.4432
27.4800
27.4412
f1 (ppm)
Intensity

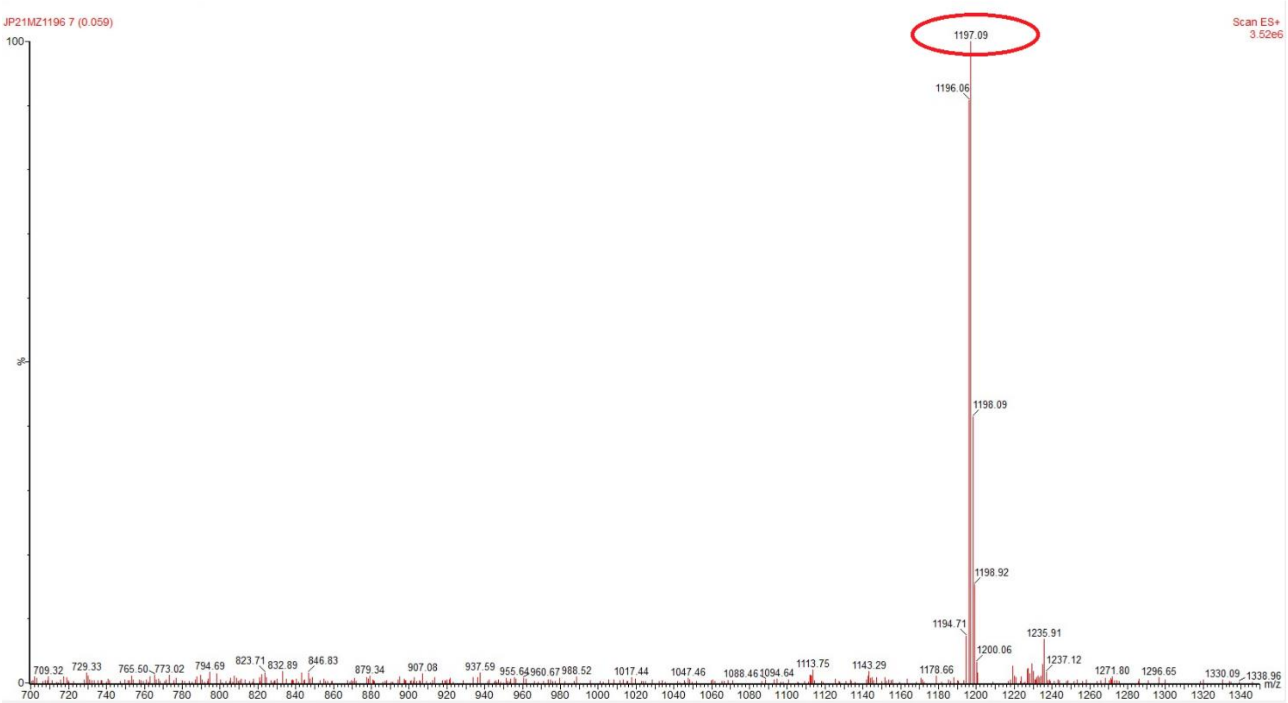

Figure S4. ^{1}H, ^{13}C NMRs and MS spectra of **V1511**

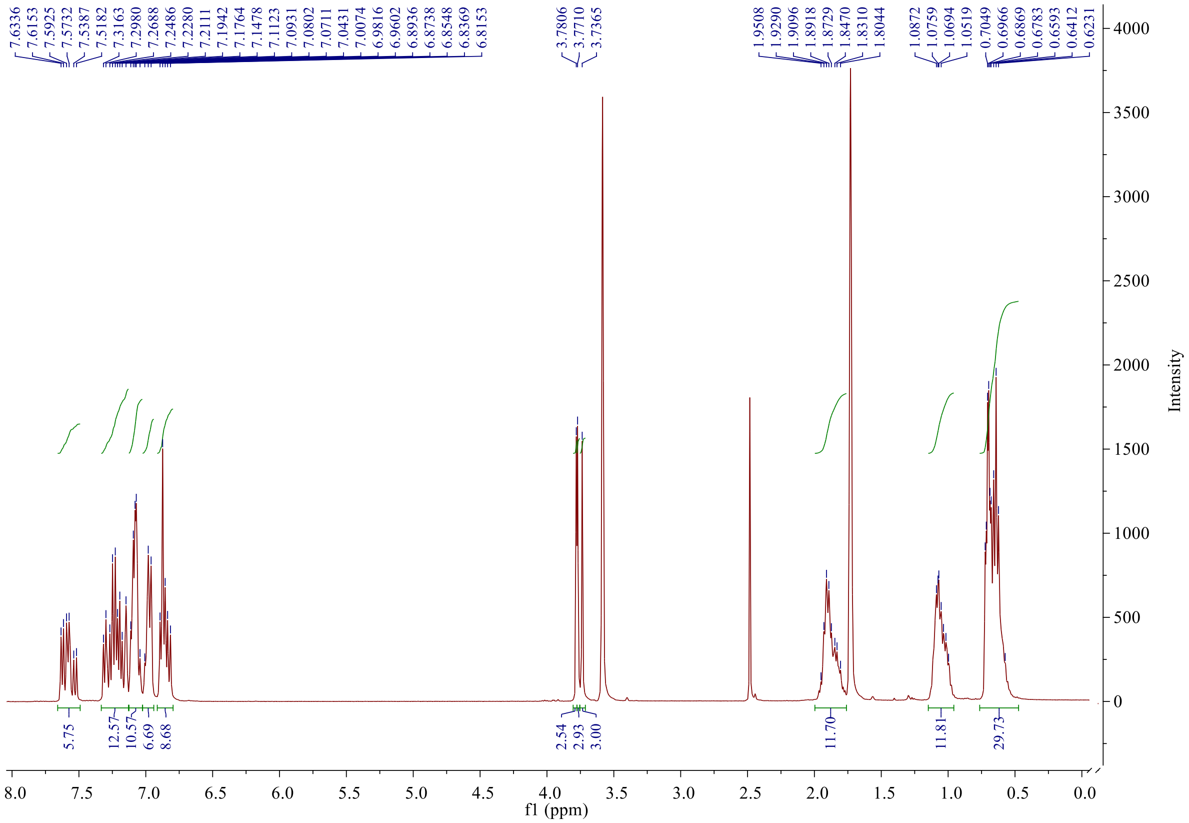

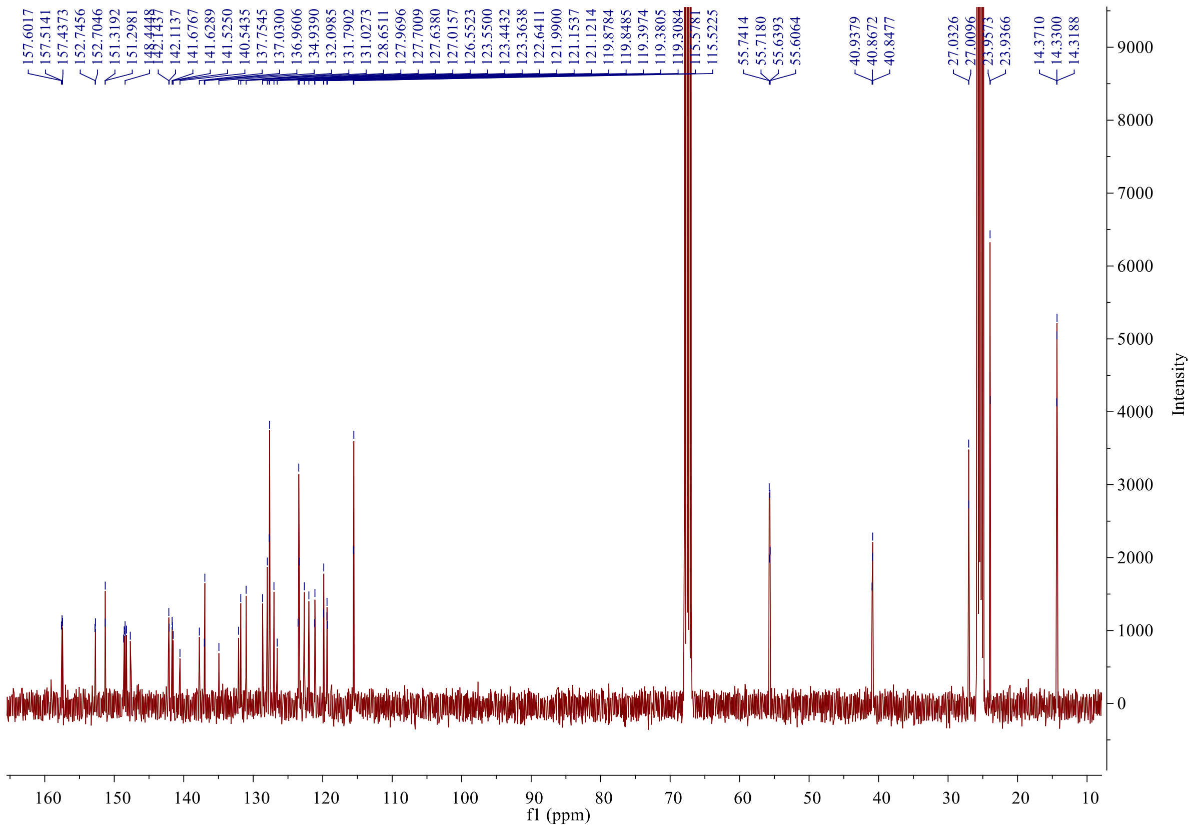

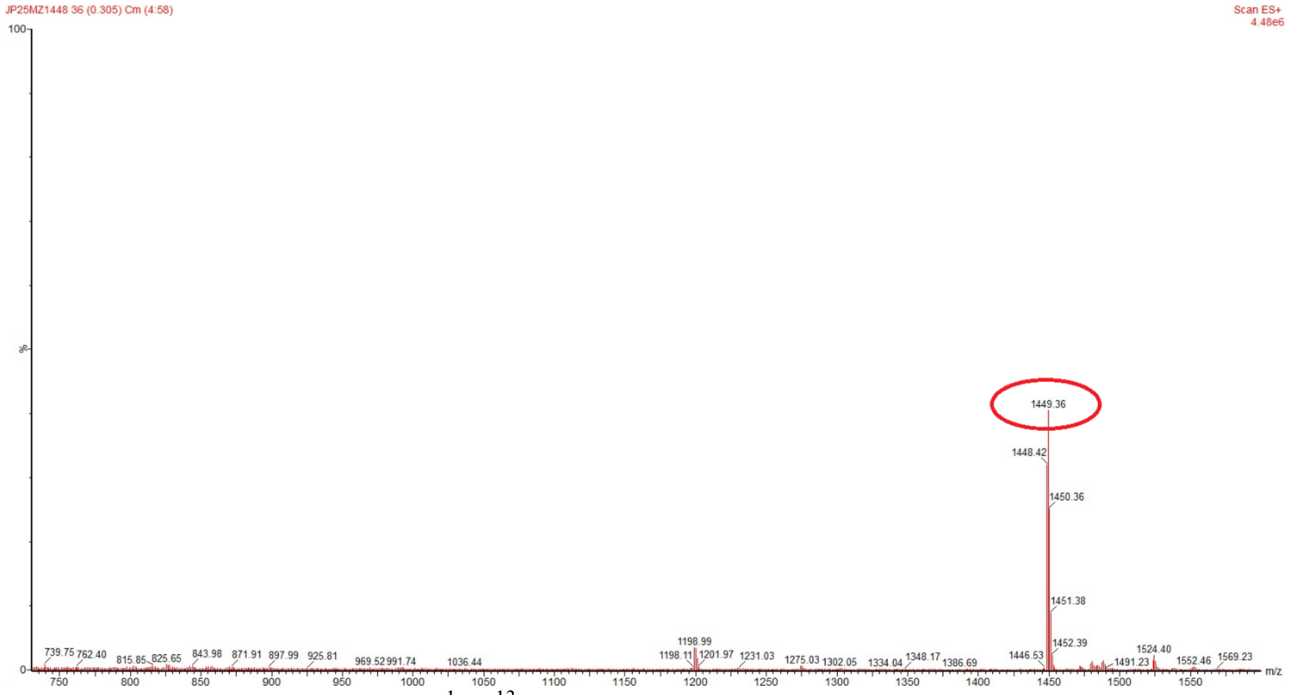

Figure S5. ^{1}H, ^{13}C NMRs and MS spectra of **V1512**

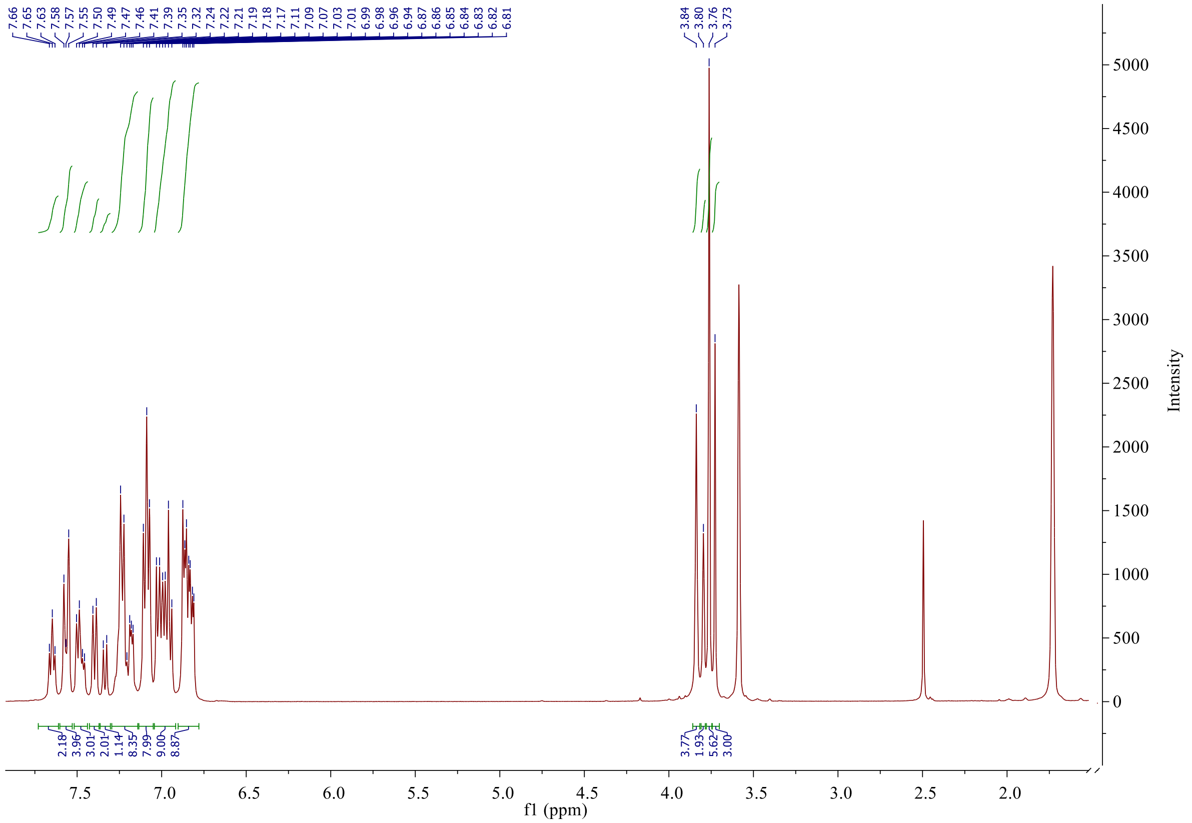

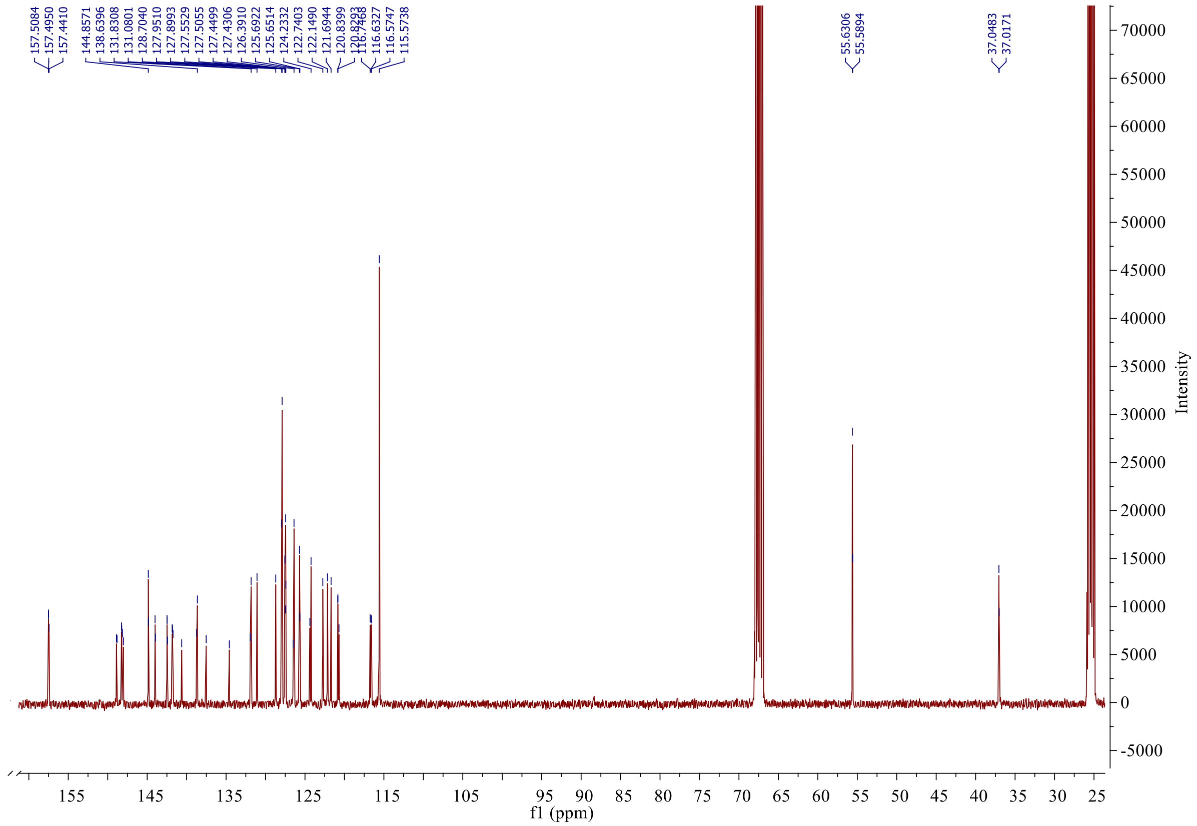

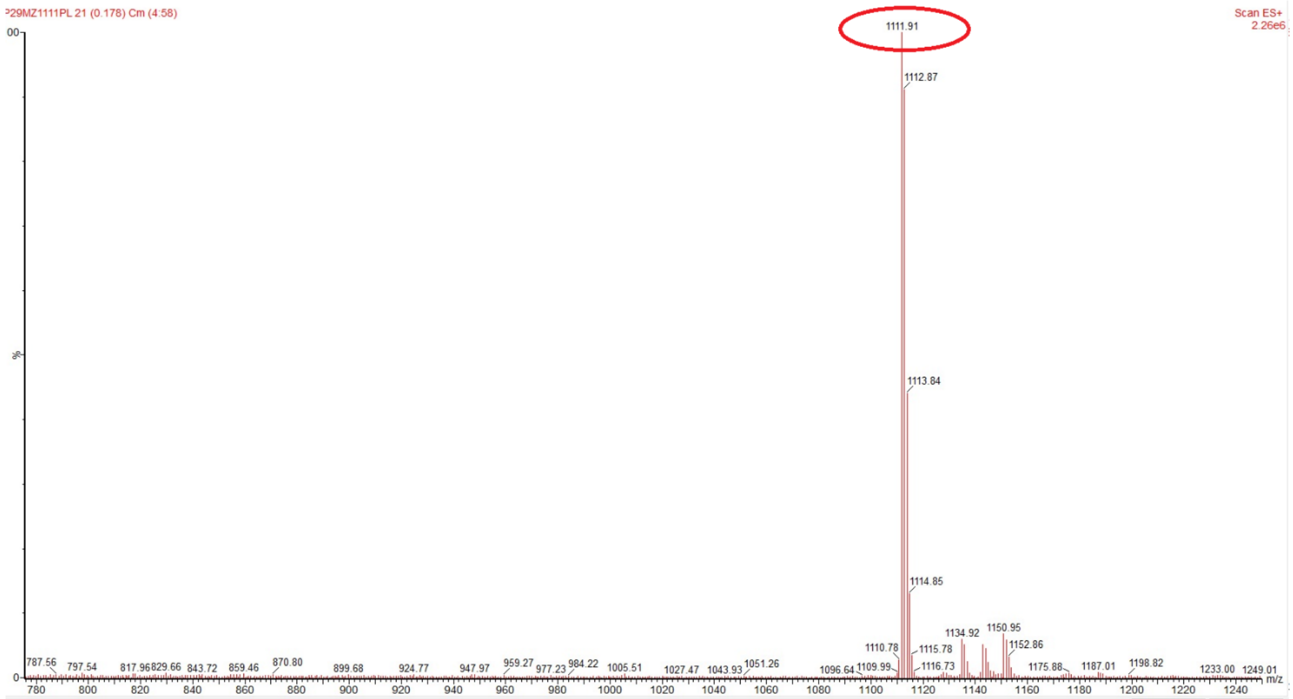

Figure S6. ^{1}H, ^{13}C NMRs and MS spectra of **V1513**

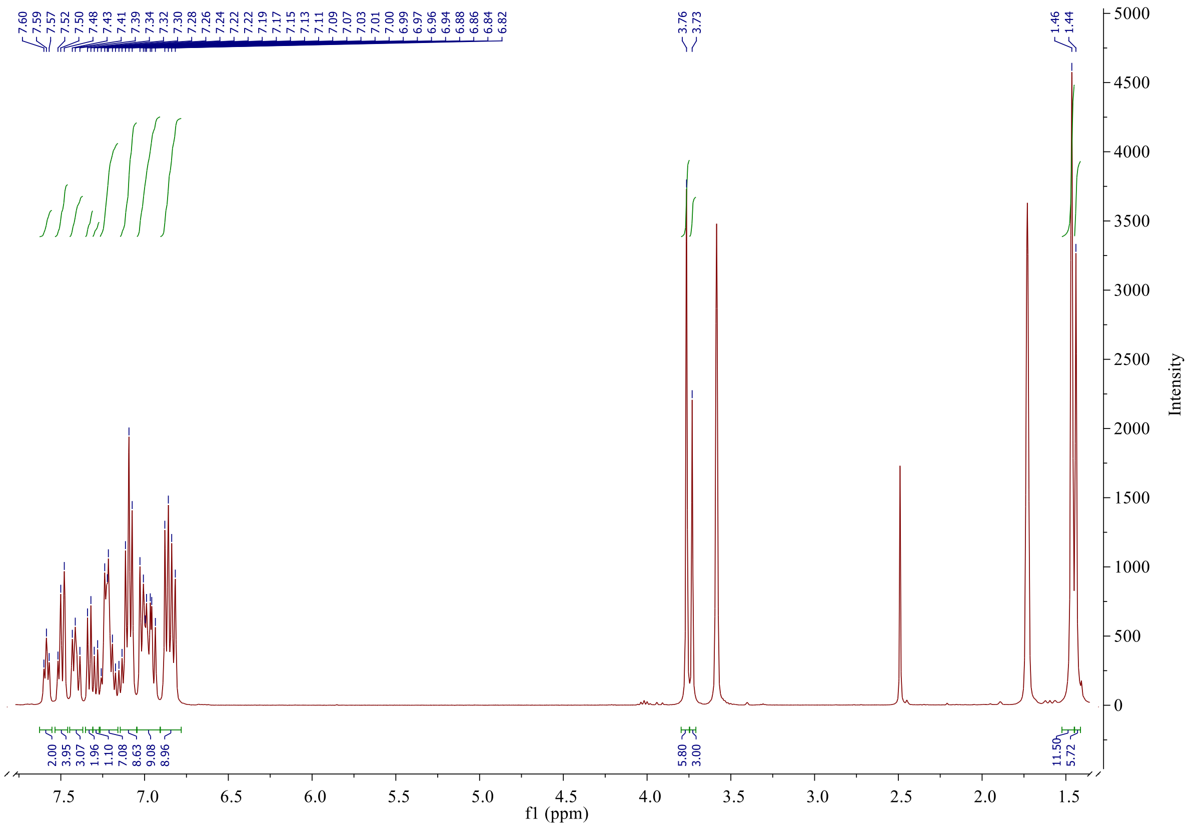

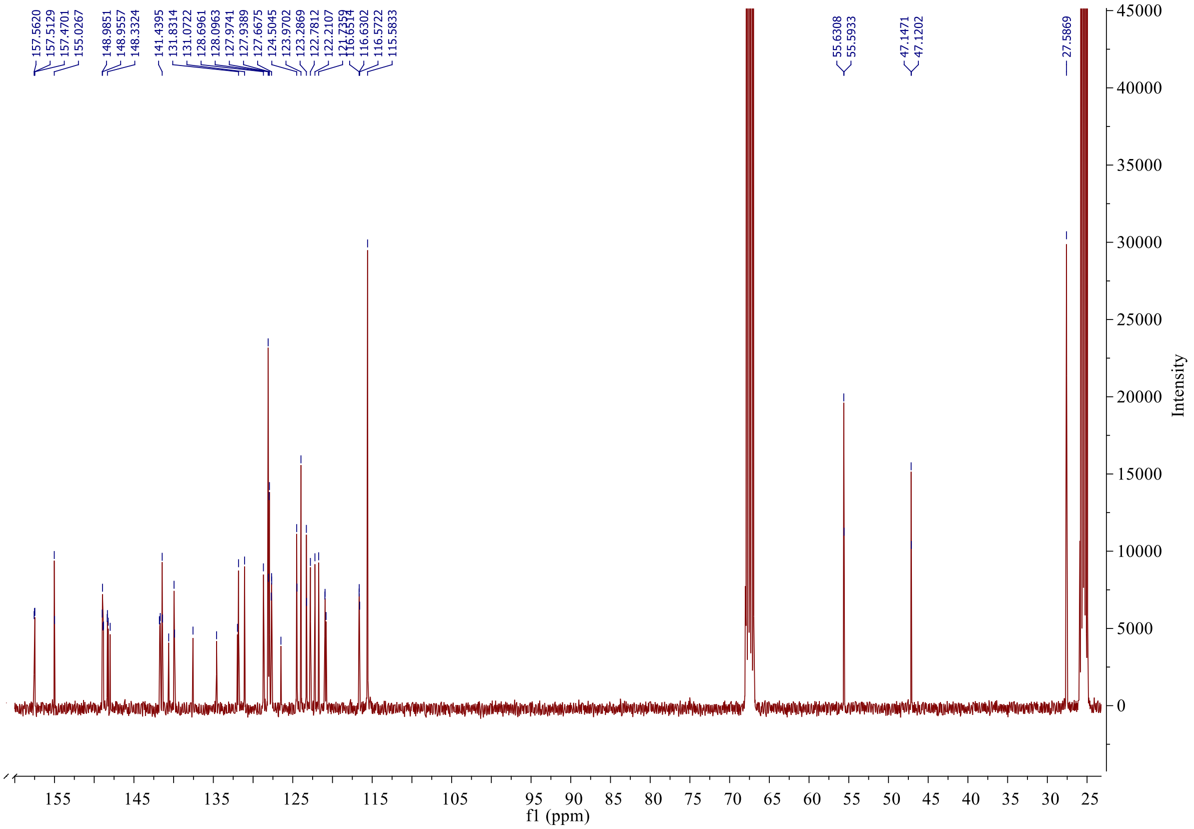

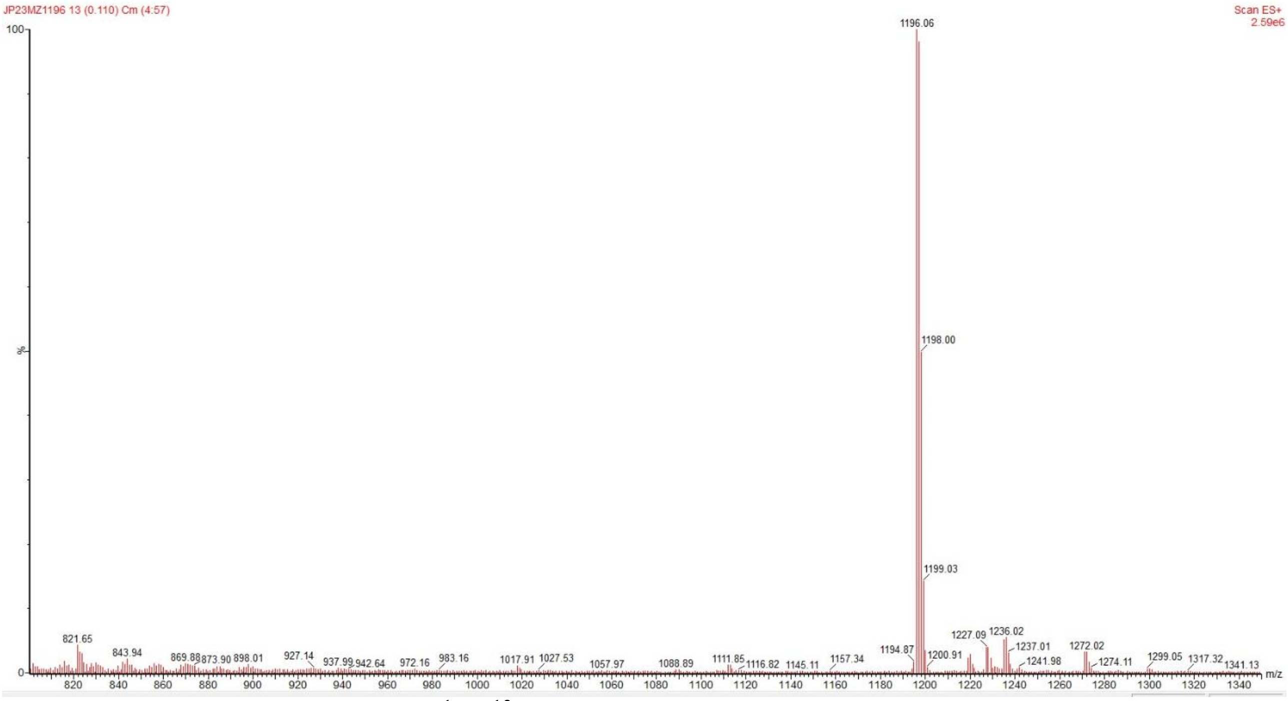

Figure S7. ^{1}H, ^{13}C NMRs and MS spectra of **V1514**

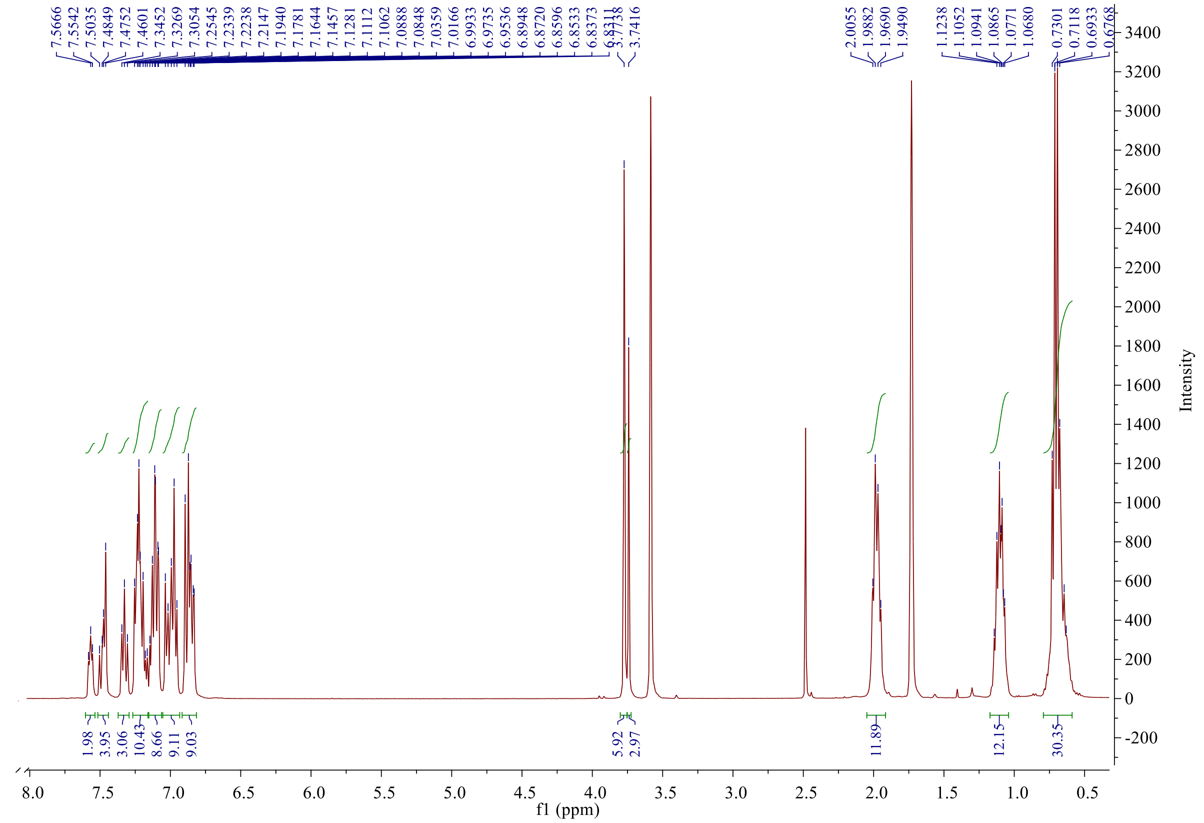
7.5666 7.5542 7.5035 7.4849 7.4752 7.4601 7.3452 7.3269 7.3054 7.2545 7.2339 7.2238 7.2147 7.1940 7.1781 7.1644 7.1457 7.1281 7.1112 7.1062 7.0888 7.0848 7.0359 7.0166 6.9933 6.9735 6.9536 6.8948 6.8720 6.8596 6.8533 6.8373 6.8311 3.7738 3.7416
2.0055 1.9882 1.9690 1.9490
1.1238 1.1052 1.0941 1.0865 1.0771 1.0680
0.7301 0.7118 0.6933 0.6768
1.98 3.95 3.06 10.43 8.66 9.11 9.03 5.92 2.97 11.89 12.15 30.35
f1 (ppm)
Intensity

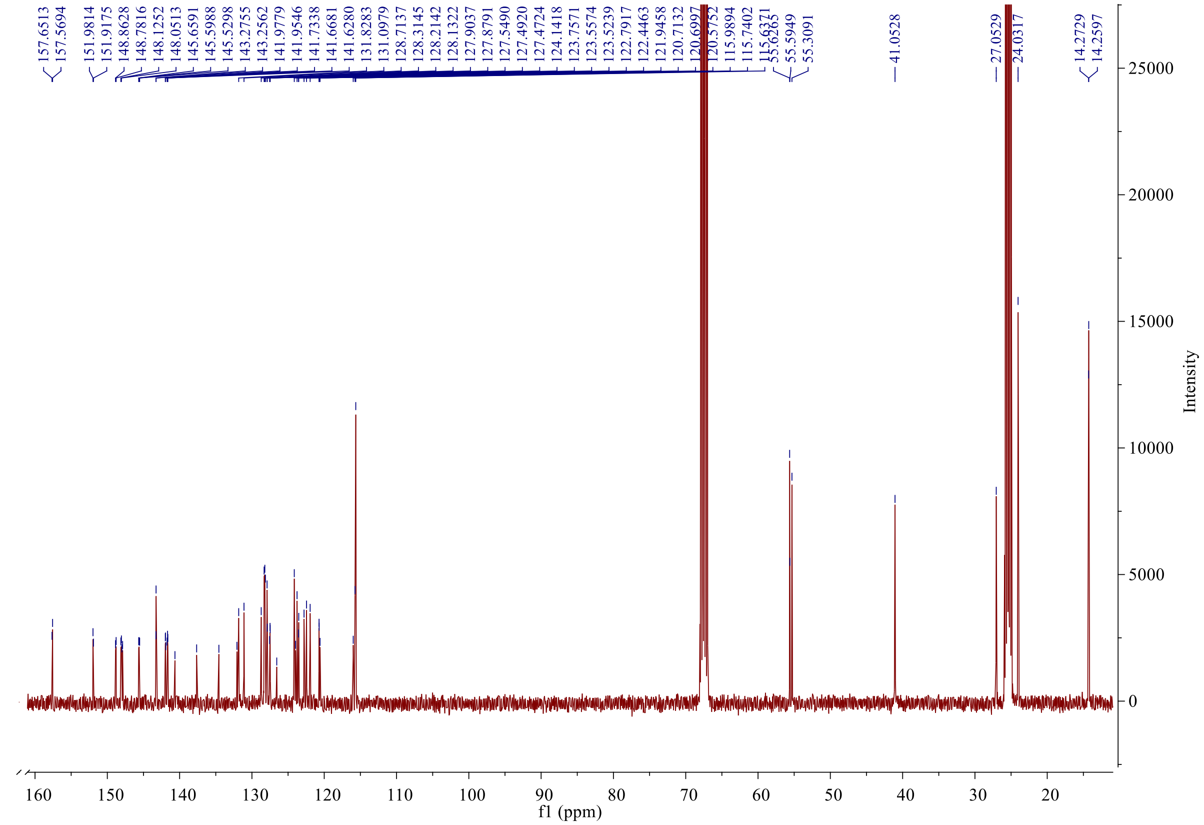
157.6513 157.5694 151.9814 151.9175 148.8628 148.7816 148.1252 148.0513 145.6591 145.5988 145.5298 143.2755 143.2562 141.9779 141.9546 141.7338 141.6681 141.6280 131.8283 131.0979 128.7137 128.3145 128.2142 128.1322 127.9037 127.8791 127.5490 127.4920 127.4724 124.1418 123.7571 123.5574 123.5239 122.7917 122.4463 121.9458 120.7132 120.6997 120.5752 115.9894 115.7402 115.6371 55.6265 55.5949 55.3091
41.0528
27.0529 24.0317
14.2729 14.2597
f1 (ppm)
Intensity

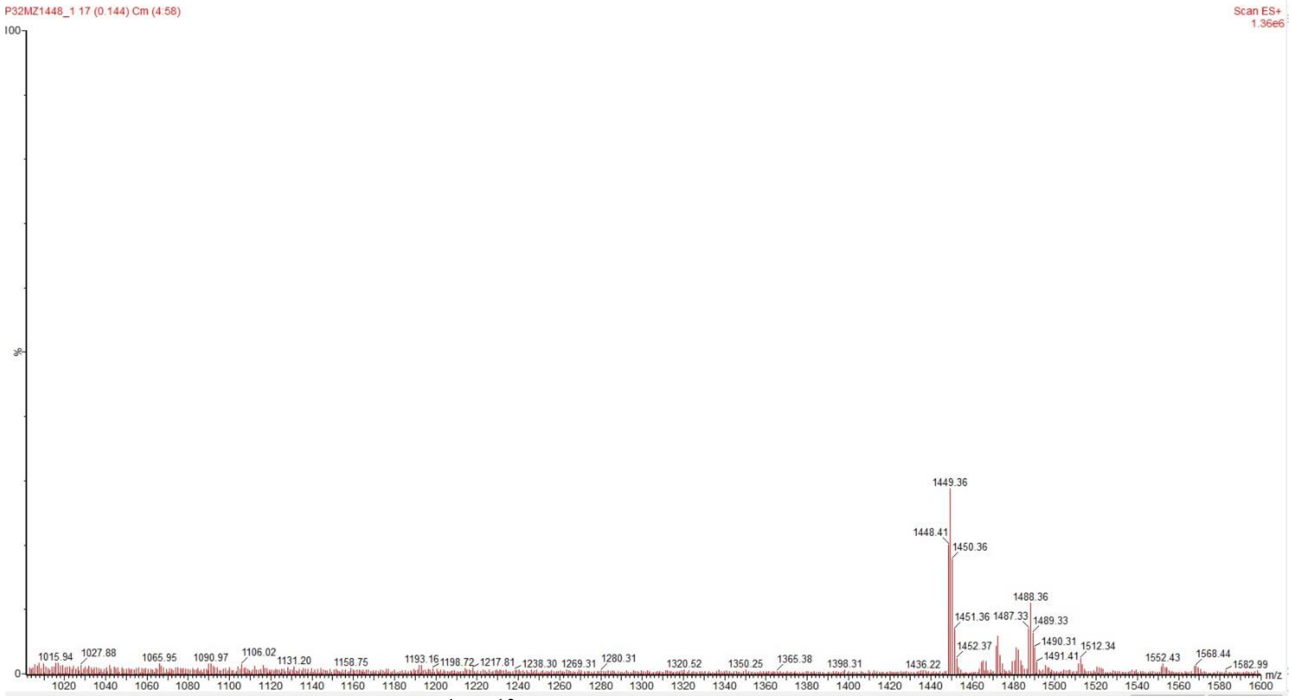

Figure S8. ^{1}H, ^{13}C NMRs and MS spectra of **V1515**

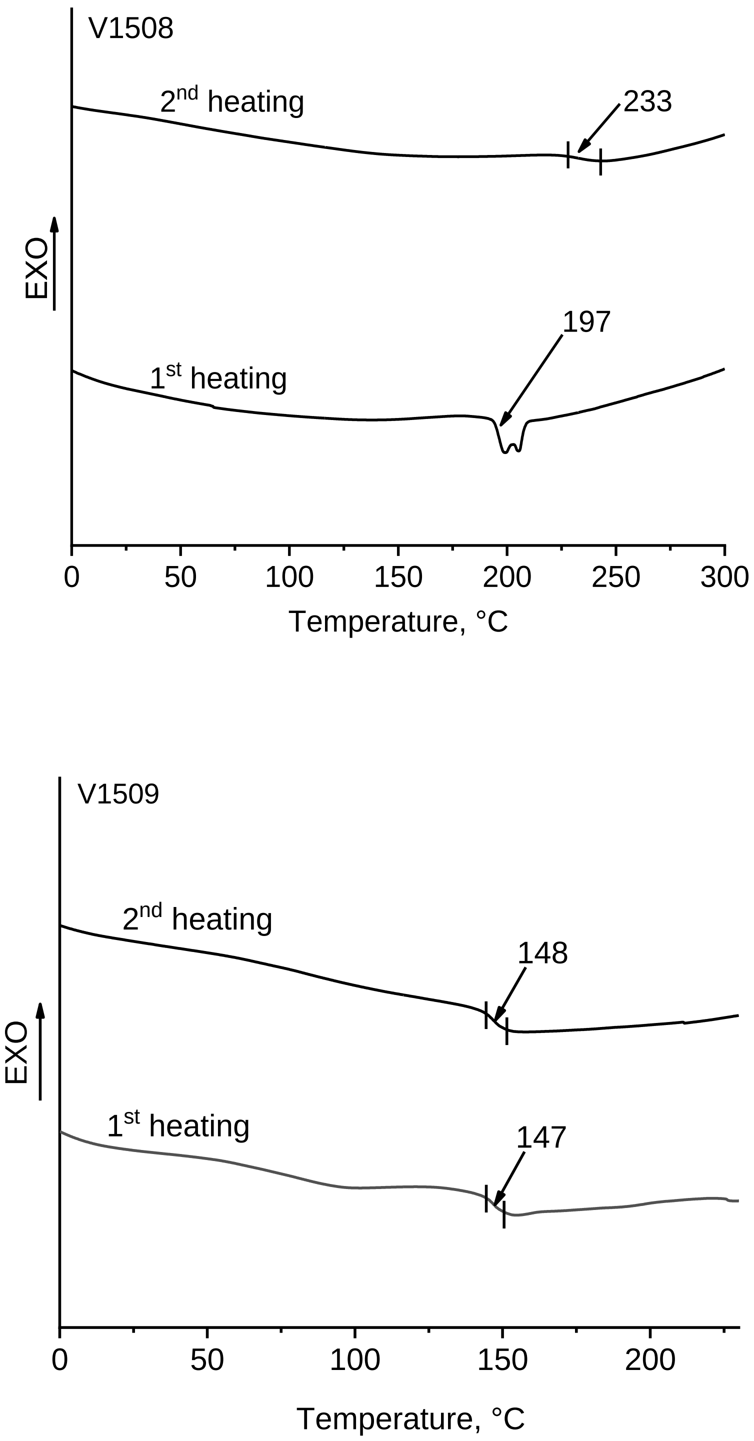

Figure S9. DSC curves of tested HTMs.

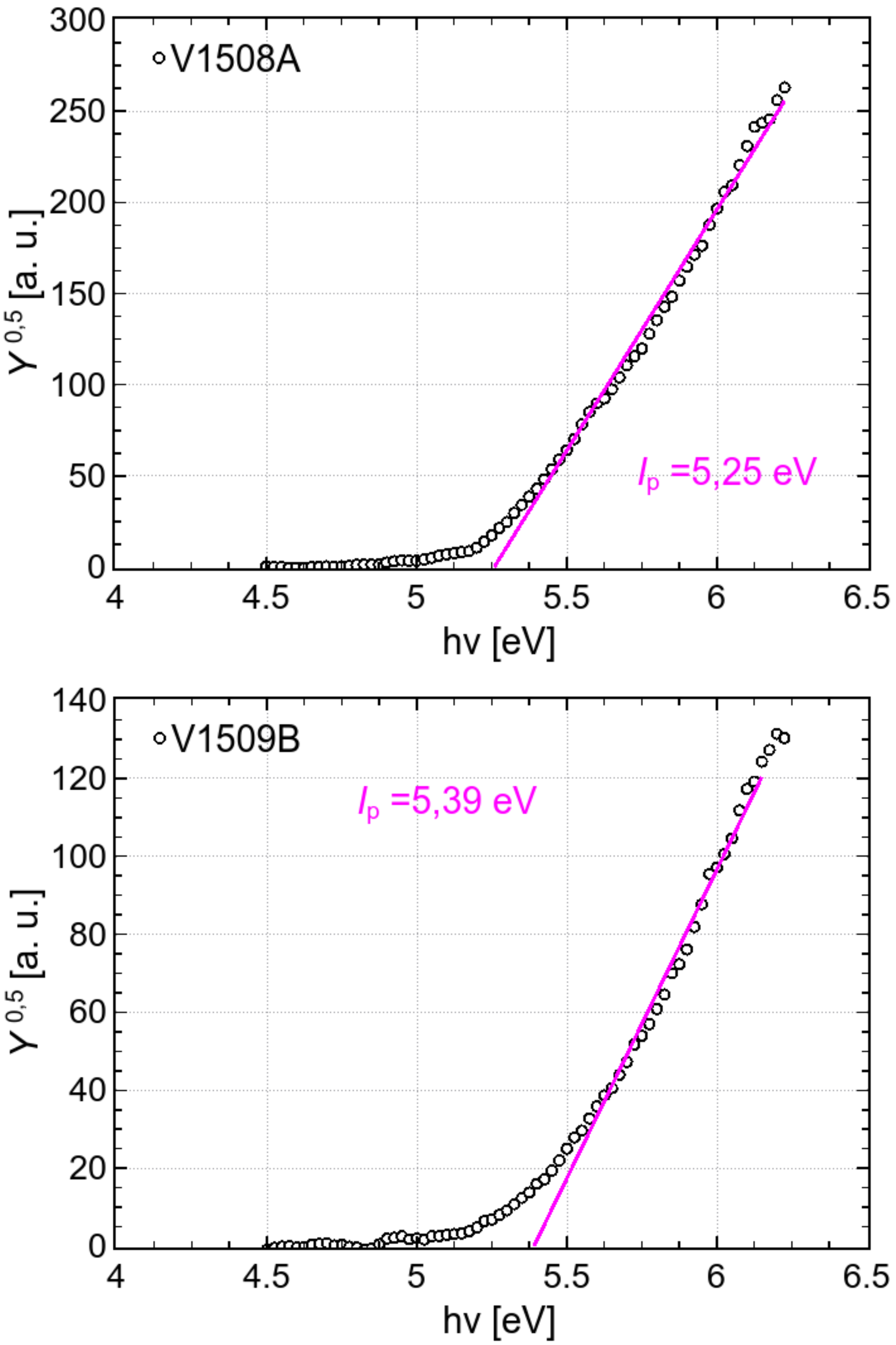

Figure S10. Photoemission in air spectra of the doped **V1508** and **V1509**.

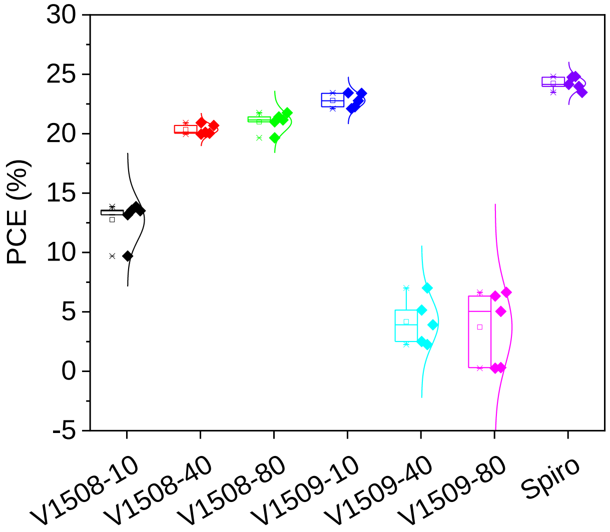

Figure S11. PCE statistic of device with different HTMs.

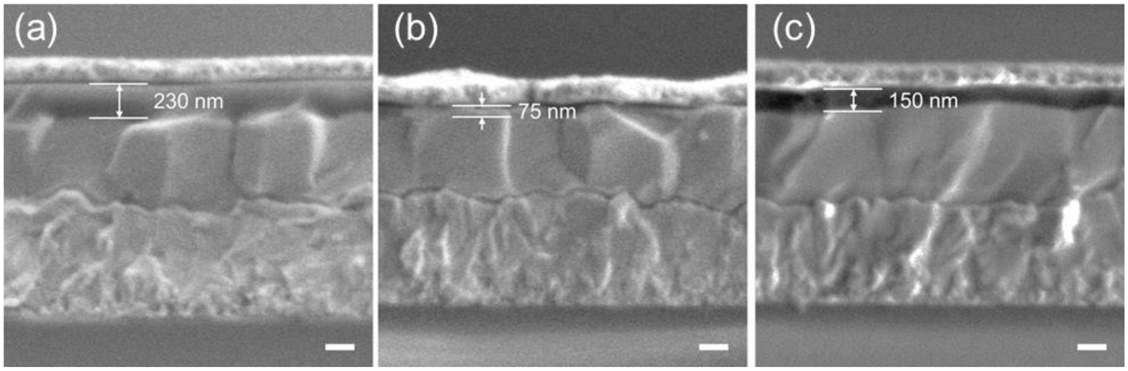

Figure S12. Cross-section SEM image of the optimal devices based on spiro-OMeTAD (a), **V1509** (b), and **V1508** (c) HTMs. Scale bars, 200 nm. The thicknesses of spiro-MeOTAD, **V1509**, and **V1508** HTMs are 230 nm, 75 nm, and 150 nm, respectively. The thickness of SnO_2, perovskite, and Au layers are ~30 nm, ~680 nm, and ~150 nm, respectively.

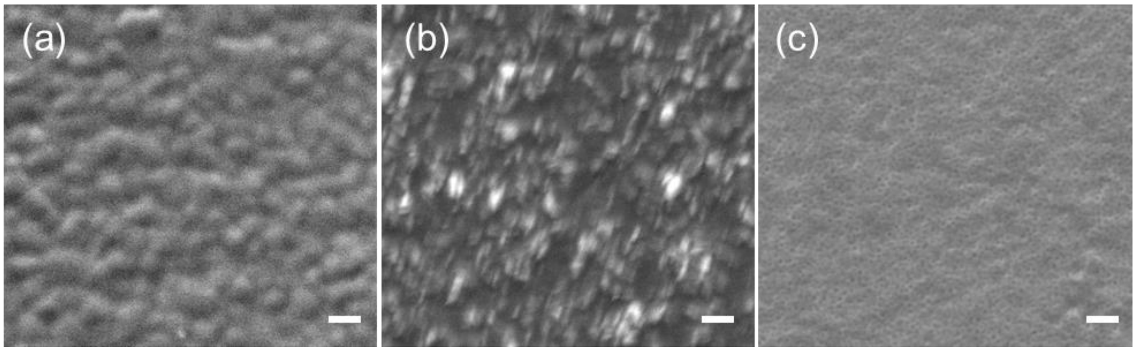

Figure S13. SEM image of the FTO/SnO_2/perovskite/HTM samples based on spiro-OMeTAD (a), **V1509** (b), and **V1508** (c) HTMs. Scale bars, 1 µm.

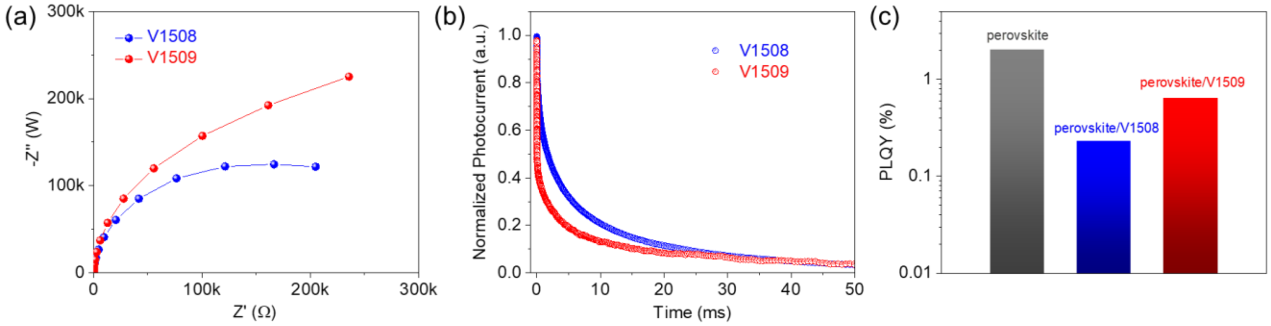

Figure S14. (a) Impedance responses of devices using different HTMs measured at a 0.8 V bias under dark conditions. (b) Transient photocurrent decays of **V1508** and **V1509**-based devices. (c) PLQY of the pure perovskite and perovskite/HTM films.

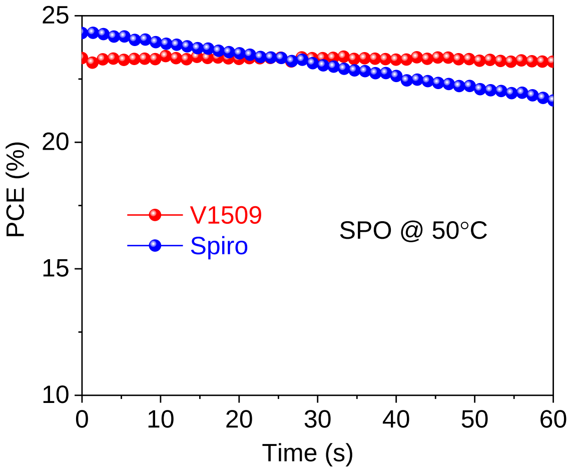

Figure S15. SPO of devices measured at 50˚C.

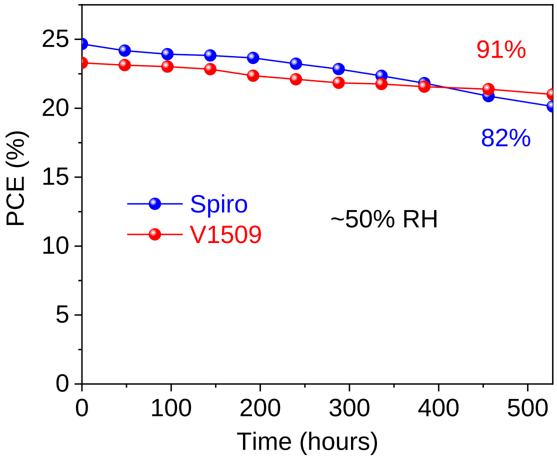

Figure S16. PCE evolution of devices under ambient of 50 RH%.

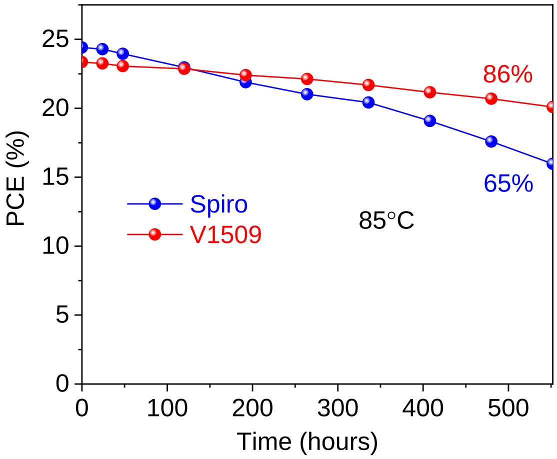

Figure S17. PCE evolution of devices at 85˚C in nitrogen.

References

1. Xu, B.; Chi, Z.; Yang, Z.; Chen, J.; Deng, S.; Li, H.; Li, X.; Zhang, Y.; Xu, N.; Xu, J. Facile Synthesis of a New Class of Aggregation-Induced Emission Materials Derived from Triphenylethylene. *J. Mater. Chem*. 2010, *20*, 4135. DOI:10.1039/C0JM00229A
2. Li, G.; Yang, L.; Liu, J.; Zhang, W.; Cao, R.; Wang, C.; Zhang, Z.; Xiao, J.; Xue, D. Light‐Promoted C–N Coupling of Aryl Halides with Nitroarenes. *Angew. Chem., Int. Ed*. 2021, 60 5230. DOI:10.1002/anie.202012877
3. Song, Y.; Xu, W.; Zhu, D. Synthesis and Properties of Cyclic Ethylene-Bridged 3, 6-fluorene Dimer and its Linear Analogues. *Tetrahedron Lett*. 2010, 51 4894. DOI:10.1016/j.tetlet.2010.07.064
4. Rakstys, K. Paek, S.; Drevilkauskaite, A.; Kanda, H.; Daskeviciute, S.; Shibayama, N.; Daskeviciene, M.; Gruodis, A.; Kamarauskas, E.; Jankauskas, V.; Getautis, V.; Nazeeruddin, M. Carbazole-Terminated Isomeric Hole-Transporting Materials for Perovskite Solar Cells. *ACS Appl. Mater. Interfaces*, 2020, *12*, 19710. DOI:10.1021/acsami.9b23495
5. Nekrasovas, J.; Gaidelis, V.; Kamarauskas, E.; Viliunas, M.; Jankauskas, V. Photoemission Studies of Organic Semiconducting Materials Using Open Geiger-Müller Counter. *J. Appl. Phys.* **2019**, 126, DOI 10.1063/1.5096070.
